# OTUB1 prevents lethal hepatocyte necroptosis through stabilization of c-IAP1 during murine liver inflammation

**DOI:** 10.1038/s41418-021-00752-9

**Published:** 2021-03-12

**Authors:** Josephin Koschel, Gopala Nishanth, Sissy Just, Kunjan Harit, Andrea Kröger, Martina Deckert, Michael Naumann, Dirk Schlüter

**Affiliations:** 1 Institute of Medical Microbiology and Hospital Epidemiology, Hannover Medical School, Hannover, Germany; 2 Institute of Experimental Internal Medicine, Otto von Guericke University Magdeburg, Magdeburg, Germany; 3 Institute of Medical Microbiology and Hospital Hygiene, Otto von Guericke University Magdeburg, Magdeburg, Germany; 4 Innate Immunity and Infection Group, Helmholtz Centre for Infection Research, Braunschweig, Germany; 5 Department of Neuropathology, Faculty of Medicine and University Hospital Cologne, University of Cologne, Cologne, Germany; 6 Cluster of Excellence RESIST (EXC 2155), Hannover Medical School, Hannover, Germany

**Keywords:** Cell death and immune response, Microbiology

## Abstract

In bacterial and sterile inflammation of the liver, hepatocyte apoptosis is, in contrast to necroptosis, a common feature. The molecular mechanisms preventing hepatocyte necroptosis and the potential consequences of hepatocyte necroptosis are largely unknown. Apoptosis and necroptosis are critically regulated by the ubiquitination of signaling molecules but especially the regulatory function of deubiquitinating enzymes (DUBs) is imperfectly defined. Here, we addressed the role of the DUB OTU domain aldehyde binding-1 (OTUB1) in hepatocyte cell death upon both infection with the hepatocyte-infecting bacterium *Listeria monocytogenes* (Lm) and D-Galactosamine (DGal)/Tumor necrosis factor (TNF)-induced sterile inflammation. Combined in vivo and in vitro experiments comprising mice lacking OTUB1 specifically in liver parenchymal cells (OTUB1^LPC-KO^) and human OTUB1-deficient HepG2 cells revealed that OTUB1 prevented hepatocyte necroptosis but not apoptosis upon infection with Lm and DGal/TNF challenge. Lm-induced necroptosis in OTUB1^LPC-KO^ mice resulted in increased alanine aminotransferase (ALT) and lactate dehydrogenase (LDH) release and rapid lethality. Treatment with the receptor-interacting serine/threonine-protein kinase (RIPK) 1 inhibitor necrostatin-1s and deletion of the pseudokinase mixed lineage kinase domain-like protein (MLKL) prevented liver damage and death of infected OTUB1^LPC-KO^ mice. Mechanistically, OTUB1 reduced K48-linked polyubiquitination of the cellular inhibitor of apoptosis 1 (c-IAP1), thereby diminishing its degradation. In the absence of OTUB1, c-IAP1 degradation resulted in reduced K63-linked polyubiquitination and increased phosphorylation of RIPK1, RIPK1/RIPK3 necrosome formation, MLKL-phosphorylation and hepatocyte death. Additionally, OTUB1-deficiency induced RIPK1-dependent extracellular-signal-regulated kinase (ERK) activation and TNF production in Lm-infected hepatocytes. Collectively, these findings identify OTUB1 as a novel regulator of hepatocyte-intrinsic necroptosis and a critical factor for survival of bacterial hepatitis and TNF challenge.

## Introduction

Upon exposure to pathogens and during inflammation, hepatocytes are sensitized to apoptosis but not necroptosis resulting in disturbance of liver function and potentially liver failure [1–3]. Among the pro-inflammatory cytokines, TNF plays a pivotal role in regulating death and cell survival of hepatocytes [1–3].

Upon binding of TNF to the TNF receptor 1 (TNFR1), a complex I consisting of TNFR1-associated via death domain (TRADD), TNFR-associated-factor (TRAF) 2/5, receptor-interacting serine/threonine-protein kinase (RIPK) 1, and cellular inhibitor of apoptosis-1 (c-IAP1) assembles. The complex I triggers the nuclear factor ‘kappa-light-chain-enhancer’ of activated B-cells (NF-κB)-dependent transcription of pro-survival genes such as B cell lymphoma-2 (Bcl-2) and cellular FLICE-like inhibitory protein (c-FLIP) [4, 5]. TNFR1 ligation may also initiate caspase-dependent apoptosis via TNFR1 complex IIa consisting of procaspase-8, TRADD, and Fas-associated protein with death domain (FADD), if NF-κB is insufficiently activated. Alternatively, a death complex IIb consisting of procaspase-8, FADD, and RIPK1 may form, which drives RIPK1-mediated apoptosis. Importantly, RIPK1 may also induce necroptosis [2, 5, 6] mediated by complex formation of RIPK1 and RIPK3, phosphorylation of the pseudokinase mixed lineage kinase domain-like protein (MLKL) by RIPK3, and the subsequent MLKL-mediated disruption of the plasma membrane. Interestingly, caspase-8 inhibits necroptosis by cleaving RIPK1, while in hepatocytes, RIPK1 may also prevent apoptosis by inhibiting degradation of the survival factor TRAF2 [7–10].

The Gram-positive bacterium *Listeria monocytogenes* (Lm) can induce cell type-dependent apoptosis and necroptosis of infected target cells in the liver. Within a few hours after systemic infection, Lm homes to the liver, infects Kupffer cells, and induces necroptosis [11]. Subsequently, released bacteria infect hepatocytes and may induce hepatocyte apoptosis [12]. In nonlethal listeriosis, bacteria are cleared from the liver by the immune response without any residual liver damage. Control of Lm in the liver is critically dependent on TNF produced by Kupffer cells, macrophages, and hepatocytes as well as TNFR1 expression of myeloid cells but not of hepatocytes [13, 14].

In contrast to listeriosis, treatment with TNF or lipopolysaccharide (LPS) in combination with D-Galactosamine (D-Gal) mediates hepatocyte apoptosis and acute liver failure [15, 16]. In this model, D-Gal inhibits hepatocyte transcription and TNF signals through TNFR1 resulting activation of caspases and apoptosis of hepatocytes [15–18]. In contrast, hepatocytes are relatively resistant to the induction of necroptosis. However, inhibition of caspase-8 and c-IAP1, respectively, can force hepatocyte necroptosis [2, 19] indicating that necroptosis is actively inhibited by yet incompletely defined mechanisms.

Post-translational modification of apoptosis and necroptosis-associated proteins by ubiquitination critically regulates these cell death pathways. Ubiquitination is a multi-step process catalyzed by ubiquitin-activating (E1), ubiquitin-conjugating (E2), and ubiquitin-ligating (E3) enzymes leading to covalent attachment of single or multiple ubiquitin molecules to target substrates. Of note, substrates linked with K48-linked polyubiquitin chains are targeted for proteasomal degradation while K63-linked or linear polyubiquitin chains confer non-degradative functions such as protein trafficking and signal transduction [20]. With respect to cell death signaling, the E3 ligase c-IAP1 prevents RIPK1-mediated apoptosis by autoubiquitination and K63-linked polyubiquitination of RIPK1 supporting activation of NF-κB instead of cell death pathways.

Ubiquitination can be reversed by deubiquitinating enzymes (DUBs). The DUB OTU domain aldehyde binding 1 (OTUB1) inhibits apoptosis in epithelial cell lines by reducing K48-linked polyubiquitination and proteasomal degradation of c-IAP1 upon stimulation with TNF-related weak inducer of apoptosis (TWEAK), which activates the CD266 (FN14) receptor [21]. We and others have demonstrated that OTUB1 preferentially reduces K48-linked polyubiquitination but can also reduce K63-linked polyubiquitination of target proteins including the suppressor of cytokine signaling 1, Smad2/3, p53, the ubiquitin-conjugating enzyme E2 N (UBE2N, UBC13), and Y-box-binding protein 1 (YB1) [22–27].

Based on the regulation of both cell death and pro-inflammatory signaling pathways by OTUB1 we explored the in vivo function of hepatocyte-specific OTUB1 during bacterial hepatitis and acute liver injury induced by DGal/LPS and DGal/TNF. Here, we demonstrate that OTUB1 prevents in vivo and in vitro Lm and TNF-driven necroptosis of hepatocytes and rescued mice from Lm and D-Gal/TNF-induced mortality. Mechanistically, OTUB1 prevented necroptosis by reducing K48-dependent proteasomal degradation of c-IAP1, thereby, enabling c-IAP1-mediated K63-linked polyubiquitination of RIPK1. Thus, OTUB1 is an endogenous inhibitor of hepatocyte necroptosis and pro-survival factor during bacterial- and TNF-induced inflammation.

## Experimental procedures

### Data reporting

No statistical methods were used to predetermine sample size.

### Mice

C57BL/6 OTUB1^FL^ [24] mice were crossed with C57BL/6 Alb-Cre mice to generate OTUB1^LPC-KO^ mice. OTUB1^FL^ mice were used as controls. C57BL/6 MLKL-deficient (MLKL^del/del^) mice [28] were crossed with OTUB1^LPC-KO^ mice to obtain MLKL^del/del^ OTUB1^LPC-KO^ mice. Age- and sex-matched 8–10 weeks mice were used for all experiments. The mice were housed under specific-pathogen-free conditions.

### Isolation of primary hepatocytes

Primary hepatocytes were isolated from mouse liver by a two-step perfusion method. First, the liver was perfused with perfusion buffer I (PBS, 10 mM HEPES, 0.05% KCl, 5 mM glucose, 0.2 mM EDTA, pH 7.4) and thereafter with pre-warmed perfusion buffer II (PBS, 20 mM HEPES, 0.05% KCl, 5 mM glucose, 1 mM CaCl_2_) containing 0.9 mg/ml collagenase (collagenase from clostridium histolyticum, Cat# C5138, Sigma-Aldrich, Steinheim, Germany). After performing a percoll gradient centrifugation, viable hepatocytes were determined using trypan blue and plated on collagen-coated plates (collagen from calf skin, Cat# C8919, Sigma-Aldrich, Steinheim, Germany) in DMEM supplemented with 10% fetal bovine serum, 1% penicillin/streptomycin, 1% L-Glutamine and 1% nonessential amino acids.

### Infection with *Listeria monocytogenes*

Mice were infected with 5 × 10^4^ colony-forming units (CFU) of *Listeria monocytogenes* wild type (Lm, EGD strain) in 200 µl of PBS (pH 7.4) intravenously.

In vitro, cells were infected with Lm (multiplicity of infection (MOI) of 10) at 37 °C for 1 h. Thereafter, extracellular Lm were killed by adding 30 µg/ml gentamicin. For analysis of the K48-linked ubiquitination, cells were infected in the presence of MG132 (10 µM, Cat# M7449, Sigma-Aldrich, Steinheim, Germany). Bacterial loads in liver and spleen were determined by plating serial dilutions of liver and spleen homogenates on brain heart infusion agar. To enumerate intracellular bacteria in cell culture, cell lysates were plated on brain heart infusion agar. Inoculated agar plates were incubated at 37 °C for 24 h and bacterial colonies were counted microscopically in a randomized blinded manner. Mice were monitored for signs of disease (weight loss, food and water intake, lethargy, activity, decline of body temperature). Mice with severe clinical signs of disease were sacrificed.

### Serology

Blood was obtained by cardiac puncture of anesthetized mice. Serum was separated by centrifugation and analyzed for alanine aminotransferase (ALT) activity and lactate dehydrogenase (LDH) release according to the manufacturer’s instructions using an ALT activity assay (Cat# MAK052-1KT, Sigma-Aldrich, Steinheim, Germany) and CyQUANT LDH Cytotoxicity Assay (Cat#C20300, Thermo Scientific, Waltham, USA), respectively.

### In vivo D-Galactosamine (DGal)/LPS and DGal/TNF models

Mice received i.p. either a combination of 400 mg/kg of DGal (Cat# 7411.1, Carl Roth, Karlsruhe, Germany) with 40 µg/kg of recombinant murine TNF (Cat# 315-01 A, PeproTech, London, UK) or 700 mg/kg DGal with *Salmonella enterica* LPS (2.5 µg/kg, Cat# L5886, Sigma-Aldrich, Steinheim, Germany) dissolved in 200 µl PBS. Mice were monitored for signs of disease (food and water intake, lethargy, activity, decline of body temperature). Mice with severe clinical signs of disease were sacrificed.

### Histopathology

Liver tissue from randomly selected mice was fixed in 4% paraformaldehyde at 4 °C. Paraffin sections were counterstained with hematoxylin and eosin (H&E).

### Necrostatin-1s and zVAD treatment

Mice were injected i.v. with either necrostatin-1s (Nec-1s, 4 mg/kg, Cat# 2263, BioVision, Inc. Milpitas, USA) or zVAD (Z-VAD-FMK, 6 mg/kg, ALX-260-020-M005, Enzo Life Sciences (Farmingdale, USA) 90 min before i.v. infection with Lm. Nec-1s and zVAD were injected daily up to day 2 p.i. and randomly selected mice were sacrificed at day 3 p.i. For survival experiments, Nec-1s administration was continued until day 5 p.i.

### HepG2 cell culture

HepG2 cells were obtained from ATCC (ATCC HB 8065). The cells were authenticated by the vendor using STR profiling prior to purchase. The cells were tested free of mycoplasma and cultured in DMEM supplemented with 10% fetal bovine serum, 1% penicillin/streptomycin, 1% L-Glutamine, and 1% nonessential amino acids. OTUB1 was deleted from the HepG2 cells by transfection with lentiviral vectors.

### Plasmids and lentivirus preparation

The shRNA plasmids were generated by inserting the hairpin oligonucleotides into lentiviral construct as previously described ([29]). Oligos contain a 21-mer sequence followed by a loop sequence and the reverse complement: OTUB1-1 (5′-GAT CCC CGC AAG TTC TTC GAG CAC TTT TCA AGA GAA AGT GCT CGA AGA ACT TCG TTT TTT GGA AG-3′) and OTUB1-2 (5′-GAT CCC CCC GAC TAC CTT GTG GTC TAT TCA AGA GAT AGA CCA CAA GGT AGT CGG TTT TTT GGA AG). Virus production was performed by calcium phosphate transfection of HEK293T cells using four plasmids encoding the helper functions (gagpol, rev, VSV-G) and the lentiviral vector. Titers were evaluated on NIH3T3 by flow cytometry.

### In vitro stimulation of HepG2 cells

HepG2 cells were stimulated with TNF (20 ng/ml, Cat# 300-01 A, PeproTech, London, UK) plus cycloheximide (CHX, 10 µg/ml, Cat# 01810, Sigma-Aldrich, Steinheim, Germany) for the indicated time points. Cells were pretreated with the inhibitors as indicated in the experiments.

### Cell death analysis

Cell death was determined by incubation of cells with 0.4% trypan blue solution (Cat# 15250061, Thermo Scientific, Waltham, USA). Samples were counted in a randomized blinded manner under a light microscope, and the percentage of nonviable blue cells was determined by the ratio of stained cells versus all cells.

### LDH assay

The LDH release was measured in serum of mice and cell culture supernatant using CyQUANT LDH Cytotoxicity Assay (Cat#C20300, Thermo Scientific, Waltham, USA) according to the manufacturer’s protocol. The measurements were carried out in a microtiter plate reader at 490 and 680 nm. Values were shown as LDH release relative to untreated mice and cells, respectively.

### Deletion of MLKL by siRNA

HepG2 cells were transfected with MLKL siRNA or negative control siRNA (Cat# L-005326-00-0010, Dharmacon, Colorado, USA). Transfection was performed with Lipofectamine RNAiMAX transfection reagent (Cat# 13778075, Thermo Scientific, Waltham, USA) according to the manufacturer’s protocol for 48 h in a six-well plate. Knockdown efficiency was controlled by western blotting (WB).

### Quantitative real-time PCR (qRT-PCR)

Total RNA was isolated from liver tissue using RNeasy Kit (Cat# 74136, Qiagen, Hilden, Germany). First strand cDNA was synthetized from 1 µg of total RNA with SuperScript Reverse Transcriptase Kit (Cat# 18064022, Thermo Scientific, Waltham, USA). qRT-PCR was performed with TaqMan probes according to the manufacturer’s protocol (Applied Biosystems, Foster City, USA) using a LightCycler 480 (Roche Diagnostics, Mannheim, Germany). All primers indicated in Supplementary Table 1 were purchased from Thermo Fisher Scientific (Waltham, USA). Gene expression was normalized to the hypoxanthine phosphoribosyltransferase as reference gene and calculated as ratio between the target gene and the reference gene employing the ΔΔCt method.

### Immune cell characterization

Liver immune cell populations were characterized by flow cytometry. Therefore, the anesthetized mice were intracardially perfused with 0.9% NaCl and the liver was subsequently minced through a 100 µm cell strainer. After erythrocyte lysis with ammonium chloride, leukocytes were isolated by Percoll density gradient centrifugation (Cat# GE17-0891-01, GE Healthcare, Uppsala, Sweden). Hepatic leukocytes were stained with mouse-specific fluorochrome-labeled antibodies as indicated in Supplementary Table 2. For detecting of intracellular cytokines, cells were re-stimulated with heat-killed Lm (80 °C, 1 h) for 5 h in the presence of Brefeldin A solution (Cat# 420601, Biolegend, San Diego, USA) followed by intracellular cytokine staining using the Intracellular Fixation & Permeabilization Kit (eBioscience by affymetrix, San Diego, USA). Samples were acquired on BD FACS Canto II (BD Biosciences, San Jose, USA) followed by data analysis using FlowJo X software (Tree Star, Ashland, USA).

### Protein lysate preparation and western blotting

Liver tissue was minced in radioimmunoprecipitation assay buffer (Cat# 9806, Cell Signaling Technology, Danvers, USA) containing protease inhibitor cocktail (Cat# P8340, Sigma-Aldrich, Steinheim, Germany), phenylmethylsulfonyl fluoride (Cat# 8553, Cell Signaling Technology, Danvers, USA) and PhosSTOP (Cat# 04 906 837 001, Roche Diagnostics Mannheim, Germany) incubated for 30 min on ice and subsequently centrifuged at 13,000 × *g* at 4 °C for 15 min. Supernatant was collected and protein concentrations were measured with Bradford assay. Equal amounts of protein were fractionated on SDS-polyacrylamide gel, blotted on a polyvinylidene fluoride membrane, and blocked at room temperature for 1 h with 5% bovine serum albumin (BSA) or 5% milk, respectively. Proteins were probed with primary antibodies as listed in Supplementary Table 3. Blots were developed using Pierce ECL 2 Western Blotting Substrate (Cat# 32132, Thermo Fisher Scientific, Waltham, USA) and images were captured with the Intas Chemo Cam Luminescent Image Analysis system (INTAS Science Imaging Instruments, Göttingen, Germany). For quantification, the LabImage Platform software was used.

### Immunoprecipitation

Protein lysates prepared as described before were pre-cleared with GammaBind G Sepharose beads (Cat# 17-0885-01, GE Healthcare, Uppsala, Sweden) at 4 °C for 1 h with agitation. Protein samples were centrifuged at 10,000 × *g* at 4 °C for 10 min to remove the beads. Equal protein amounts were incubated with the respective primary antibody overnight on a rocking platform at 4 °C, followed by 2 h incubation with the beads at 4 °C. The beads were collected by centrifugation at 10,000 × *g* at 4 °C for 30 s and subsequently washed for three times with cold PBS. Immune complexes were eluted with 2 × lane marker reducing sample buffer (Cat# 39000, Thermo Scientific, Waltham, USA) at 99 °C for 5 min. To remove the beads, samples were centrifuged and western blot was performed as described above.

### Immunofluorescence

HepG2 cells plated on a coverslip were infected with Lm or stimulated with TNF/CHX as described before. Following stimulation, cells were fixed in 4% paraformaldehyde for 10 min at room temperature and thereafter permeabilized with 0.1% Triton-X-100 for 7 min. Nonspecific bindings were blocked by incubating the cells for 45 min in 0.5% BSA, 0.3% Triton-X-100, 5% sucrose, 10% fetal calf serum) before staining the cells with anti-p-MLKL (Cat# ab187091, Abcam, Cambridge, UK) overnight on a rocking platform. The slides were washed three times with PBS and then incubated with the fluorochrome-conjugated secondary antibody (Goat Anti-Rabbit IgG, Alexa Fluor Plus 594, Thermo Fisher Scientific, Waltham, USA) for 2 h protected from light. After washing with PBS, the slides were mounted on a glass slide using ProLong Diamond Antifade with DAPI (Cat# P36962, Thermo Fisher Scientific, Waltham, USA). Images were captured on Microscope Axio Imager Z1 (Carl Zeiss AG, Oberkochen, Germany).

### Statistical analysis

All graphs represent the mean; error bars indicate the standard error of the mean (SEM). The statistical significance was determined with the software Prism 8 (GraphPad Software, Inc., San Diego, USA) using the respective test as indicated in the figure legends. All experiments were performed at least twice.

## Results

### Liver parenchymal cell-specific OTUB1 protects mice from bacterial and DGal/LPS-induced mortality

**Fig. 1:**
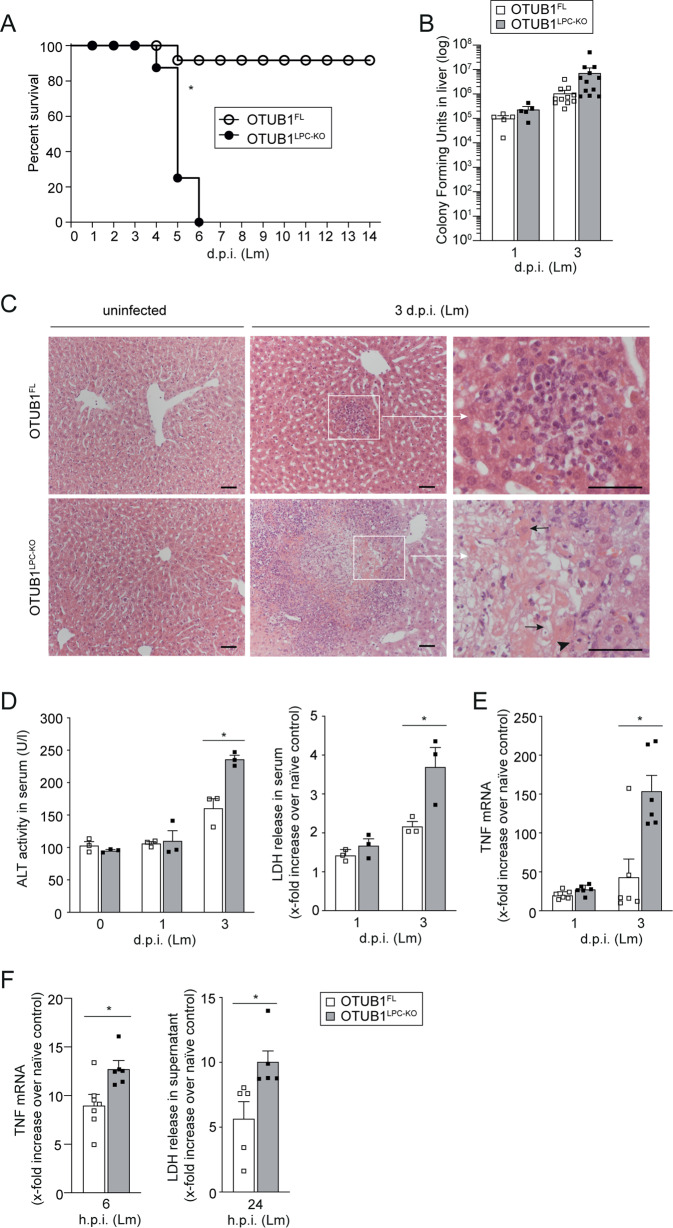
Hepatocyte-specific OTUB1 expression protects from lethal listeriosis. **A**–**E** OTUB1^LPC-KO^ and OTUB1^FL^ mice were i.v. infected with 5 × 10^4^ CFU of Lm. **A** Survival was monitored daily up to day 14 after Lm infection (*n* = 16 per group). **B** Bacterial loads were determined in the livers of Lm-infected OTUB1^LPC-KO^ and OTUB1^FL^ mice at day 1 (*n* = 5 per group) and 3 p.i. (*n* = 11 per group). Data of individual mice and the mean + SEM are shown. **C** Representative histopathological images of uninfected and Lm-infected (3 d.p.i.) livers from OTUB1^LPC-KO^ and OTUB1^FL^ mice (*n* = 3 mice per group and time point). In an infected OTUB1^LPC-KO^ mouse central necrosis containing cellular debris is surrounded by inflammatory cells (lower middle panel, a white quadrat marks the border necrosis). In the right lower image, a high power magnification of the necrotic lesion is shown. Arrows point to uninfected swollen hepatocytes with pale nuclei and eosinophilic cytoplasm. Arrowhead indicates a swollen hepatocyte with pale cytoplasm. In an infected OTUB1^FL^ mice (upper middle panel), hepatocytes are viable and only small inflammatory foci are present (marked by a white quadrant). In the right upper image, a high power magnification of the small inflammatory lesion is shown. (H&E, scale bars indicate 50 µm). **D** ALT activity (left panel) and LDH release (right panel) were measured in serum samples of Lm-infected OTUB1^LPC-KO^ and OTUB^FL^ mice at the indicated time points (*n* = 3 per group and time point). **E** Relative gene expression of TNF mRNA was quantified by qRT-PCR. Bars show the mean fold increase over the respective uninfected mouse strain + SEM (*n* = 6 mice per group). **F** Cultured hepatocytes isolated from OTUB1^LPC-KO^ and OTUB1^FL^ mice were infected with Lm (MOI of 10) for 6 and 24 h, respectively. Cells were harvested and analyzed for the relative TNF mRNA expression by qRT-PCR (left panel, *n* = 6–7). Bar graphs show the mean fold increase + SEM over the respective uninfected controls. After 24 h of infection, the LDH release was measured in the supernatant (right panel, *n* = 5). In **B** and **D**–**F**, data of individual mice and the mean + SEM are shown, *p ≤ 0.05. **A** Log-rank test, **B** Kruskal–Wallis test, **C**, **E**, **F** Student’s *t* test, *p ≤ 0.05. CFU colony-forming units, Lm *Listeria monocytogenes*, p.i. post infection, qRT-PCR quantitative real-time PCR, H&E haematoxylin & eosin, ALT alanine aminotransferase, LDH lactate dehydrogenase, MOI multiplicity of infection, h hour.

To investigate the function of OTUB1 in the liver, we generated OTUB1^LPC-KO^ mice lacking OTUB1 expression specifically in liver parenchymal cells (Fig. S1A). OTUB1^LPC-KO^ mice developed normally and liver architecture was regular without inflammation up to 1 year (Fig. S1B–D). To study the function of hepatocyte-specific OTUB1 during infection, OTUB1^LPC-KO^ and OTUB1^FL^ mice were i.v. infected with Lm. While all OTUB1^LPC-KO^ mice succumbed up to day 6 p.i., 90% of OTUB1^FL^ mice survived (Fig. 1A). At day 3 p.i., bacterial loads in liver and spleen did not differ significantly between both strains of mice (Figs. 1B and S1E) indicating that the protective effect of OTUB1 on survival was independent of pathogen control.

OTUB1^LPC-KO^ mice developed severe listeriosis characterized by large inflammatory foci with severely damaged hepatocytes and central necrosis surrounded by leukocytes (Fig. 1C). In contrast, control mice exhibited numerous small inflammatory foci containing viable hepatocytes but no necrosis throughout the hepatic parenchyma (Fig. 1C). OTUB1 limited liver dysfunction and cell death in Lm-infected mice as indicated by lower serum levels of ALT and LDH (Fig. 1D), respectively, in OTUB1^FL^ mice. Hepatocyte OTUB1 expression did not impact on the total number of inflammatory leukocytes recruited to the liver and hepatic transcription of interleukin (IL)−6, IL-1β, and interferon (IFN)γ (Fig. S1F, G). However, hepatic TNF mRNA expression was significantly increased in the livers of OTUB1^LPC-KO^ mice at day 3 p.i. (Fig. 1E). Thus, OTUB1 prevented lethal listeriosis, liver necrosis, and production of TNF.

Next, we identified the cell population producing increased amounts of TNF in the absence of OTUB1 during listeriosis. As shown in Fig. S1H, TNF production of hepatic CD45^+^ F4/80^+^ CD11b ^low^ Ly-6C ^low^ Kupffer cells, CD45^+^ F4/80^+^ CD11b ^int^ Ly-6C ^high^ inflammatory monocytes and CD45^+^ F4/80^+^ CD11b ^high^ Ly-6C ^int^ monocyte-derived macrophage was comparable in both Lm-infected mouse strains. However, upon in vitro Lm infection of hepatocytes, TNF mRNA expression and LDH release (Fig. 1F) were significantly increased in OTUB1-deficient hepatocytes. In agreement with the in vivo data (Fig. 1B), bacterial loads were comparable between cultivated Lm-infected hepatocytes of both mouse strains (Fig. S1I) suggesting that OTUB1 may suppress TNF production by hepatocytes without affecting pathogen control.

**Fig. 2:**
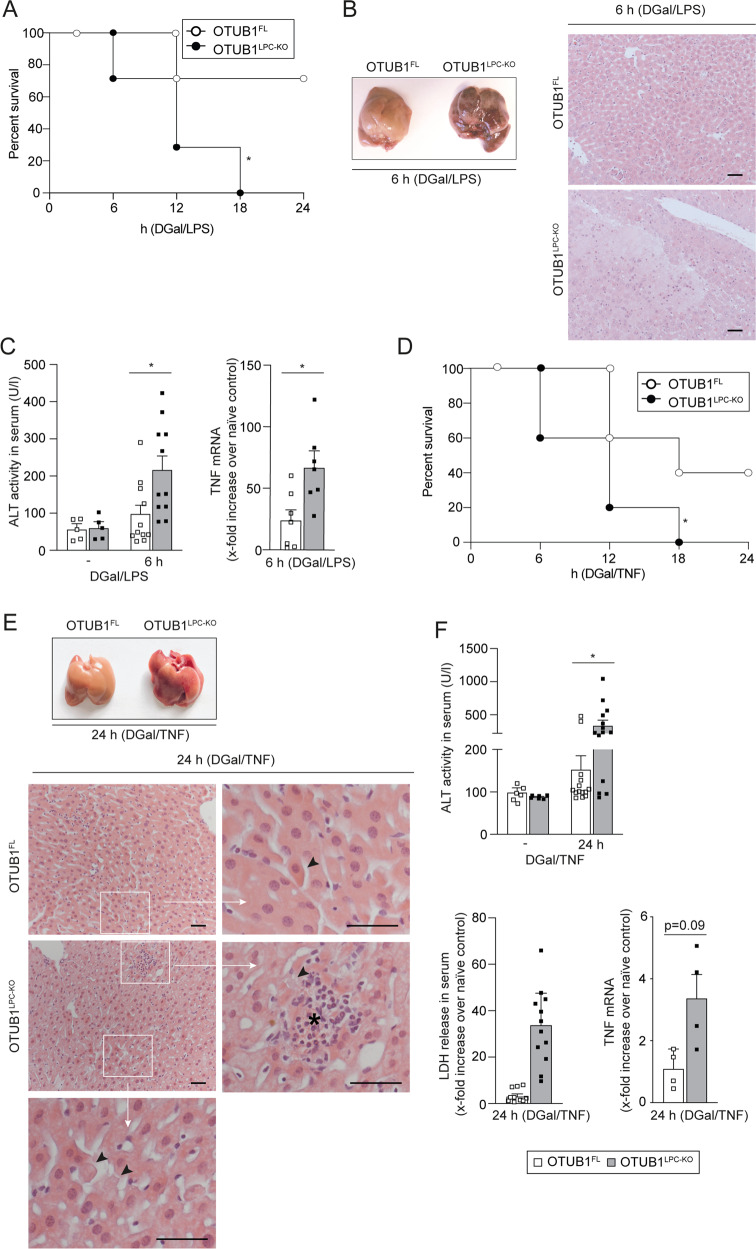
OTUB1^LPC-KO^ mice are highly susceptible to DGal/LPS and DGal/TNF challenge. **A**–**C** OTUB1^LPC-KO^ and OTUB1^FL^ mice were challenged i.p. with DGal (700 mg/kg)/LPS (2.5 µg/kg). **A** Survival rates were monitored up to 24 h post DGal/LPS challenge (*n* = 7 per group). **B** Macroscopic (left panel) and histopathological (right panel) examinations of OTUB1^LPC-KO^ and OTUB1^FL^ livers at 6 h post DGal/LPS-treatment. Representative images are depicted. In an OTUB1^LPC-KO^ mouse, sharply demarcated necrosis is present which was absent from liver of a OTUB1^FL^ mouse (H&E, scale bars indicate 50 µm). **C** At 6 h post DGal/LPS challenge, serum was collected and analyzed for ALT levels (left panel, unstimulated: *n* = 5, stimulated: *n* = 11 mice per group). Quantitative RT-PCR analysis for the relative TNF mRNA expression in liver tissue after 6 h of DGal/LPS injection (right panel, *n* = 7 mice per group). In **D**–**F** OTUB1^LPC-KO^ and OTUB1^FL^ mice were challenged i.p. with DGal (400 mg/kg)/TNF (40 µg/kg). **D** Survival was determined up to 24 h post DGal/TNF treatment (*n* = 5 mice per group). **E** Representative macroscopic (left panel) and histopathological (right panel) images of DGal/TNF-treated OTUB1^LPC-KO^ and OTUB1^FL^ livers (24 h post challenge). In the liver of a OTUB1^LPC-KO^ mouse, a small inflammatory infiltrate (asterisk) is associated with a damaged hepatocyte (arrowhead, upper white quadrant). High power magnifications of the marked areas of the liver of an OTUB1^LPC-KO^ mouse depicts multiple damaged hepatocytes with swollen and eosinophilic cytoplasm and an absence of nuclei (arrowheads). On the contrary, liver architecture of an OTUB1^FL^ mouse is normal. High power magnification of the liver of a OTUB1^FL^ mouse shows only single hepatocytes with swollen and eosinophilic cytoplasm (arrowhead) H&E, scale bars represent 50 µm). **F** Serum samples were analyzed for ALT activity (upper panel) and LDH release (lower left panel) at 24 h post DGal/TNF treatment (ALT unstimulated: *n* = 6 mice per group: all other groups: *n* = 14). Quantitative RT-PCR analysis (lower right panel) were performed for TNF mRNA expression (*n* = 4). Mean + SEM are displayed, **C**, **F** Student’s *t* test, *p ≤ 0.05. DGal D-Galactosamine, LPS lipopolysaccharides, H&E haematoxylin & eosin, TNF tumor necrosis factor, mRNA messenger ribonucleic acid, qRT-PCR quantitative real-time PCR, ALT alanine aminotransferase, LDH lactate dehydrogenase.

To further study the role of OTUB1 in TNF responses of hepatocytes, we employed the DGal/LPS model, which induces TNF-dependent hepatocyte death and acute liver failure. Upon i.p. injection of low dose LPS in combination with DGal, all OTUB1^LPC-KO^ mice succumbed up to 24 h p.i, whereas 70% of OTUB1^FL^ mice survived (Fig. 2A). As early as 6 h after DGal/LPS injection, the liver of OTUB1^LPC-KO^ mice harbored large necrosis evident already macroscopically, which were absent in the liver in OTUB1^FL^ mice (Figs. 2B and S2A). Histologically, sharply demarcated hepatic necrosis was present in OTUB1^LPC-KO^ but not in OTUB1^FL^ mice (Figs. 2B and S2A). Serum ALT was significantly increased in OTUB1^LPC-KO^ mice. Additionally, OTUB1^LPC-KO^ mice expressed significantly more TNF mRNA in the liver illustrating an augmented pathogen-independent TNF production in the absence of OTUB1 (Fig. 2C). To directly assess the role of TNF in the enhanced hepatocyte death in OTUB1^LPC-KO^ mice, we substituted LPS by TNF and challenged mice with equally low amounts of TNF in combination with DGal. In accordance to the DGal/LPS experiments, survival of OTUB1^LPC-KO^ mice was significantly reduced as compared to OTUB1^FL^ mice (Fig. 2D). Macroscopically, livers of OTUB1^LPC-KO^ mice showed liver damage and hemorrhages, which were absent in OTUB1^FL^ mice (Figs. 2E and S2B). OTUB1^LPC-KO^ hepatocytes were swollen, the cytoplasm was eosinophilic and nuclei were no longer identified (Figs. 2E and S2B). Small inflammatory infiltrates were associated with damaged hepatocytes in OTUB1^LPC-KO^ mice (Figs. 2E and S2B) In contrast, livers of OTUB1^FL^ mice were histologically normal (Fig. 2E) or showed only moderate liver pathology (Fig. S2B). Furthermore, serum ALT and LDH were elevated and hepatic TNF mRNA was increased in OTUB1^LPC-KO^ mice (Fig. 2F). Taken together, these data indicate that OTUB1 prevents TNF-induced hepatocyte death in vivo.

### OTUB1 reduced ERK activation and shifted cell death signaling in *Listeria*-infected and DGal/TNF-stimulated mice

**Fig. 3:**
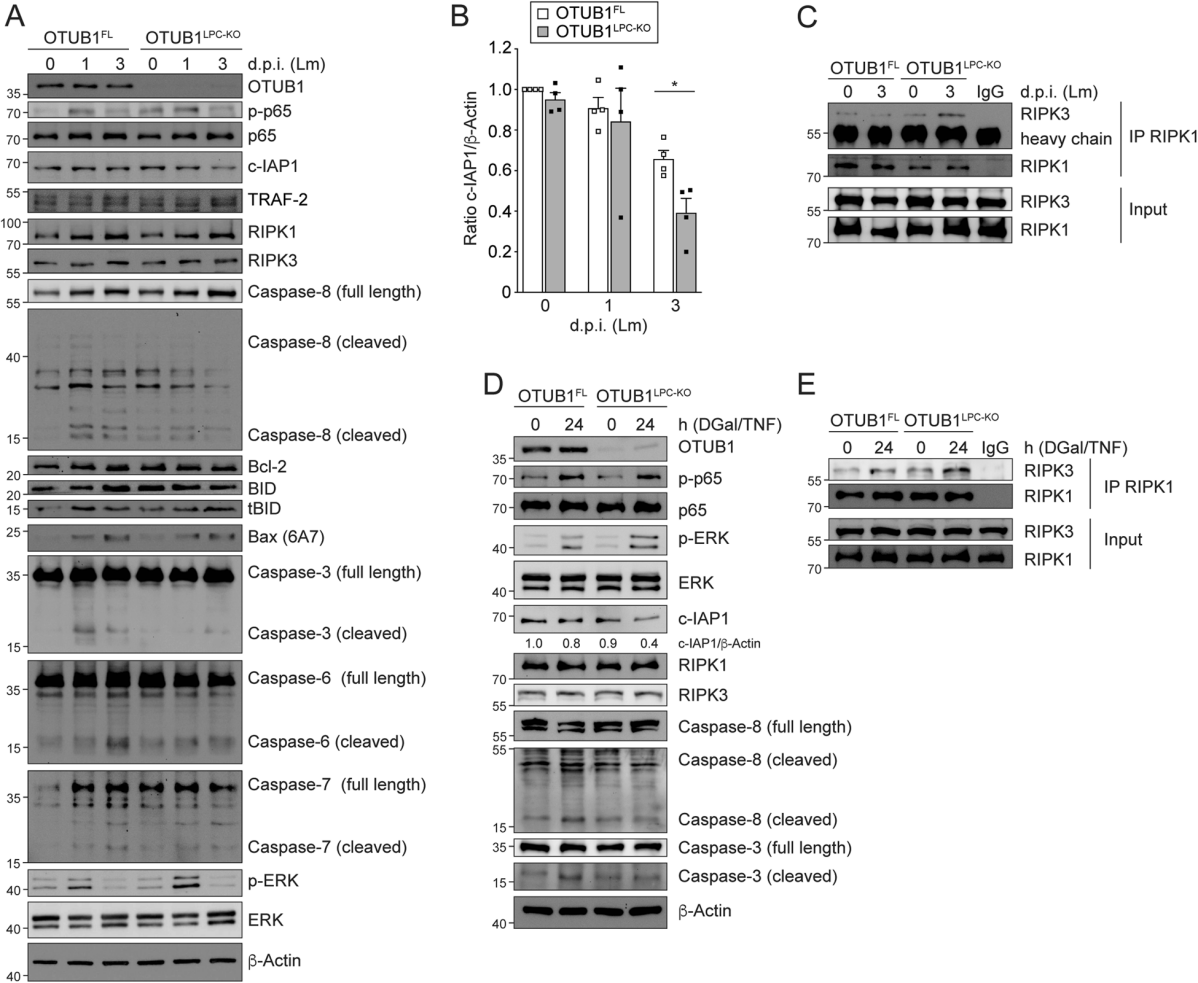
OTUB1 prevents degradation of c-IAP1 and necrosome formation. **A**–**C** OTUB1^LPC-KO^ and OTUB1^FL^ mice were i.v. infected with 5 × 10^4^ CFU of Lm. **A** Whole liver lysates were harvested at day 1 and 3 p.i., and analyzed for the indicated proteins by WB. **B** c-IAP1 expression was quantified based on WB data and normalized to β-actin. Data of individual mice and the mean ratio + SEM of c-IAP1/β-Actin are shown (*n* = 4 per group and time point), (Student’s *t* test, *p ≤ 0.05). **C** Protein lysates isolated from uninfected and Lm-infected OTUB1^LPC-KO^ and OTUB1^FL^ mice (day 3 p.i.) were immunoprecipitated with anti-RIPK1 antibody and subsequently blotted with anti-RIPK1 and anti-RIPK3 antibodies. **D**, **E** DGal (400 mg/kg)/TNF (40 µg/kg) were injected i.p. into OTUB1^LPC-KO^ and OTUB1^FL^ mice. After 24 h, protein lysates were prepared from unstimulated and stimulated total liver tissue. **D** Protein lysates were assessed for OTUB1, p65, p-p65, ERK, p-ERK, c-IAP1, RIPK1, RIPK3, caspase 3 and caspase 8 expression by WB. **E** RIPK1-complexes were immunoprecipitated from unstimulated and DGal/TNF-stimulated livers using anti-RIPK1. Immunoprecipitates were probed with anti-RIPK1 and anti-RIPK3. All blots are representative for one of three mice per group. CFU colony-forming units, Lm *Listeria monocytogenes*, c-IAP1 cellular inhibitor of apoptosis 1, TRAF-2 TNFR-associated factor 2, RIPK receptor-interacting serine/threonine kinase, Bcl-2 B cell lymphoma-2, BID BH3-interacting-domain death agonist, tBID truncated BID, p phospho, ERK extracellular-signal-regulated kinase, d.p.i. days post infection, IP immunoprecipitation.

Since OTUB1 has been shown to inhibit TWEAK-induced apoptosis and pro-inflammatory signaling [21, 23, 30], we investigated the impact of hepatocyte-specific OTUB1 expression on NF-κB and cell death pathways. WB analysis of *Listeria*-infected liver tissue of OTUB1^LPC-KO^ and OTUB1^FL^ mice revealed an equal phosphorylation of p65 indicating a similar activation of the pathway (Fig. 3A). Additionally, the NF-κB regulated pro-apoptotic gene Bcl-2-associated x protein (Bax) (Figs. 3A and S3A) as well as the antiapoptotic genes Bcl-2 (Figs. 3A and S3B) and c-FLIP (Fig. S3C) showed equal mRNA expression levels in the livers of both genotypes.

Next, we studied the expression of c-IAP1, apoptosis, and necroptosis regulating signaling molecules in livers of *Listeria*-infected and D-Gal/TNF challenged mice, respectively. In Lm-infected mice, c-IAP1 proteins were decreased in OTUB1^LPC-KO^ mice (Fig. 3A, B). Of note, transcription of c-IAP1 mRNA was not affected following infection with *Listeria* (Fig. S3D) indicating that OTUB1 regulated c-IAP1 protein stability. In contrast, TRAF2, an inhibitor of cell death [31] was equally expressed in both genotypes (Fig. 3A).

Apoptotic cell death is tightly regulated by caspase-8, which either directly initiates apoptosis by cleaving the effector caspases-3, 6, and 7 or indirectly via cleaving BH3-interacting-domain death antagonist (BID) to tBID (truncated BID) resulting in Bax and effector caspases activation [2]. In OTUB1^FL^ mice, hepatic cleavage of procaspase-3, 6, and 7 was increased as compared to OTUB1^LPC-KO^ mice, while levels of BID and tBID were comparable between both groups (Fig. 3A). Thus, the mild liver pathology of *Listeria*-infected OTUB1-competent mice was associated with effector caspase cleavage, whereas the lethal liver pathology and hepatocyte death of OTUB1^LPC-KO^ mice was independent of the cleavage of the executioner caspases.

Since decreased c-IAP1 levels in conjunction with caspase-8 inhibition promote necroptosis [19], we analyzed RIPK1/RIPK3 necrosome formation. RIPK1 and RIPK3 were slightly increased upon infection in the livers of uninfected and Lm-infected OTUB1^FL^ and OTUB1^LPC-KO^ mice (Fig. 3A). RIPK1 immunoprecipitates from livers of both mouse strains contained RIPK3 with an increased amount in OTUB1^LPC-KO^ mice (Fig. 3C) indicating that OTUB1-deficiency increases necroptosis during listeriosis. Furthermore, we analyzed the activation of extracellular signal-regulated kinase (ERK), p-38, c-Jun N-terminal kinase (JNK), and the noncanonical NF-κB pathway. Interestingly, phosphorylation of ERK was more pronounced in the livers of OTUB1^LPC-KO^ mice as compared to OTUB1^FL^ mice, while activation of p-38, JNK, and the noncanonical NF-κB pathway did not differ between the two groups of mice (Figs. 3A and S3E).

Upon DGal/TNF injection, OTUB1 also stabilized c-IAP1 expression, slightly increased cleavage of caspase-8 and -3, suppressed RIPK1/RIPK3 complex formation, and reduced ERK phosphorylation (Fig. 3D, E) in the livers of OTUB1^FL^ as compared to OTUB1^LPC-KO^ mice. Taken together, OTUB1 stabilized c-IAP1, suppressed ERK phosphorylation and prevented *Listeria*- and TNF-induced necroptosis.

### Inhibition of RIPK1 and MLKL but not of caspases rescued liver function and prevented death of *Listeria*-infected OTUB1^LPC-KO^ mice

**Fig. 4:**
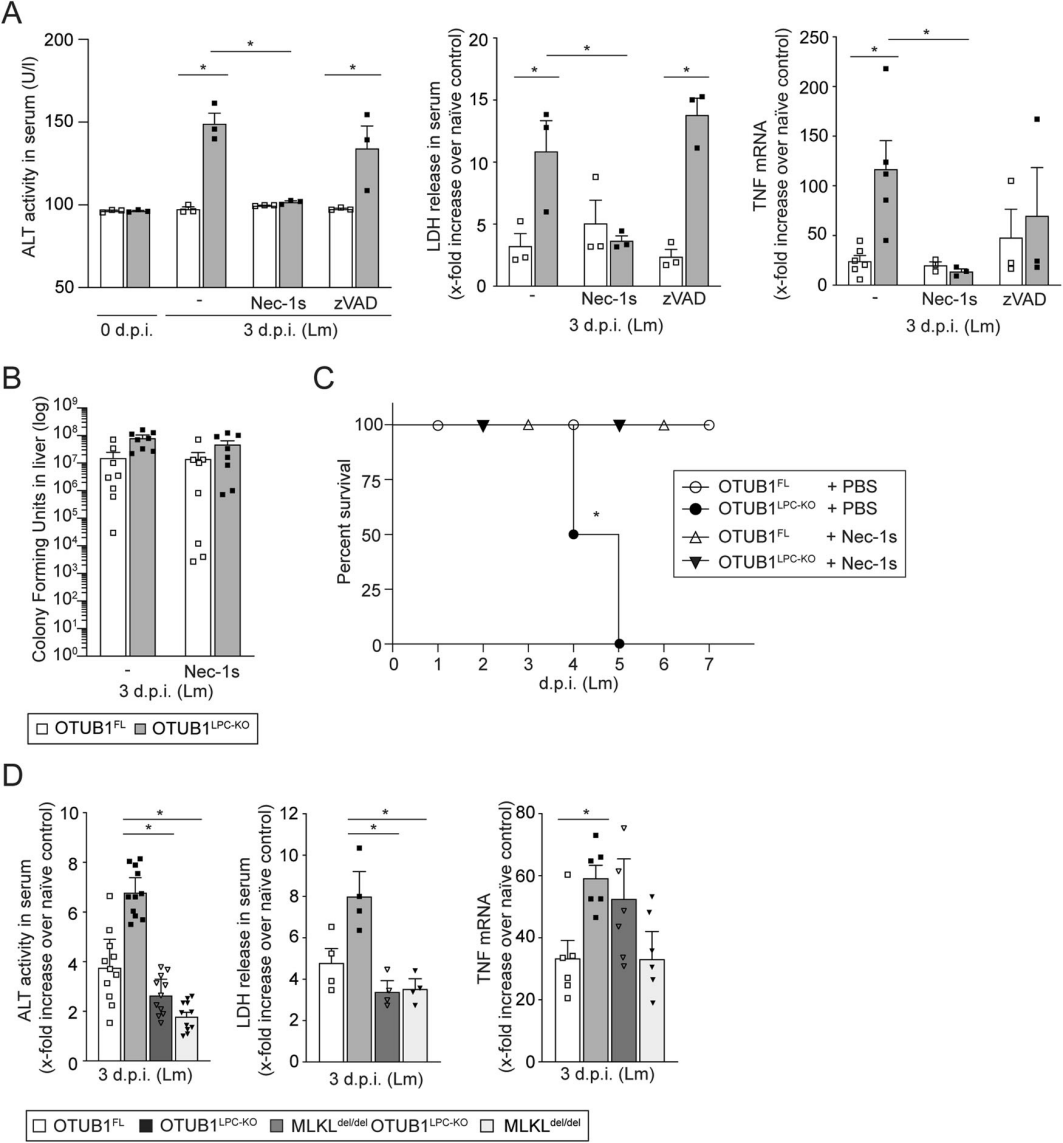
Nec-1s administration and genetic deletion of MLKL ameliorate listeriosis in OTUB1^LPC-KO^ mice. **A** Before i.v. infection with 5 × 10^4^ CFU of Lm, OTUB1^LPC-KO^, and OTUB1^FL^ mice received daily i.v. injections of Nec-1s (4 mg/kg), zVAD (6 mg/kg) and PBS, respectively, starting 90 min prior to and up to d 2 p.i. Uninfected mice and Lm-infected mice at day 3 p.i. were sacrificed, serum was collected and analyzed for ALT activity (left panel, *n* = 3) and LDH release (middle panel, *n* = 3). Hepatic expression of TNF mRNA was determined using qRT-PCR (right panel, *n* = 3–6). **B** CFUs were determined in Nec-1s-treated and PBS-treated OTUB1^LPC-KO^ and OTUB1^FL^ mice at day 3 p.i. (*n* = 8). **C** Lm-infected mice received daily administration of Nec-1s and PBS, respectively, up to day 5 p.i. The survival rates were monitored daily up to day 7 p.i. (*n* = 4 per group). **D** OTUB1^FL^, OTUB1^LPC-KO^, MLKL^del/del^, and MLKL^del/del^ OTUB1^LPC-KO^ mice were i.v. infected with 5 × 10^4^ CFU of Lm. **D** ALT activity (left panel, *n* = 11) and the release of LDH (middle panel, *n* = 4) were measured in serum samples from uninfected and Lm-infected mice (day 3 p.i.) of the indicated groups. qRT-PCR analysis was performed for hepatic TNF mRNA expression in uninfected and Lm-infected (3 d.p.i.) liver tissue (right panel, *n* = 6). Data represent mean values + SEM. **A**, **D** Two-way Anova, **B** Kruskal–Wallis test, *p ≤ 0.05. MLKL mixed lineage kinase domain-like protein, Nec-1s Necrostatin-1s, zVAD Z-VAD-FMK, PBS phosphate-buffered saline, CFU colony-forming units, Lm *Listeria monocytogenes*, p.i. post infection, ALT alanine aminotransferase, LDH lactate dehydrogenase, TNF tumor necrosis factor, qRT-PCR quantitative real-time PCR.

Since RIPK1 kinase activity can mediate both caspase-8-dependent apoptosis and RIPK1/RIPK3/MLKL-dependent necroptosis [32], we treated *Listeria*-infected OTUB1^LPC-KO^ and OTUB1^FL^ mice with the RIPK1 kinase inhibitor Nec-1s and the pan-caspase inhibitor zVAD. Nec-1s but not zVAD treatment resulted in a significant decline in serum ALT, LDH levels and hepatic TNF mRNA expression in OTUB1^LPC-KO^ mice and abolished differences between both genotypes (Fig. 4A). However, Nec-1s treatment had no influence on the bacterial burden (Fig. 4B). In accordance with the reduced liver damage and TNF production, Nec-1s treatment conferred protection against *Listeria*-induced lethality in the OTUB1^LPC-KO^ mice (Fig. 4C).

Previous reports identified MLKL as the terminal executor of necroptosis by inducing cell membrane disruption [33, 34]. Thus, we crossed OTUB1^LPC-KO^ mice with MLKL-deficient mice to obtain hepatocytes deficient in both OTUB1 and MLKL (MLKL^del/del^ OTUB1^LPC-KO^ mice, Fig. S3A). Upon infection, deletion of MLKL in OTUB1^LPC-KO^ mice significantly reduced ALT and LDH levels as compared to OTUB1^LPC-KO^ (Fig. 4D) illustrating that necroptosis aggravated liver damage in the absence of OTUB1. Of note, TNF mRNA production was equally increased in both MLKL^del/del^ OTUB1^LPC-KO^ and OTUB1^LPC-KO^ mice as compared to OTUB1^FL^ and MLKL^del/del^ mice, respectively, (Fig. 4D) indicating that OTUB1-mediated suppression of TNF mRNA is upstream of MLKL. These in vivo data suggest that hepatocyte-intrinsic OTUB1 inhibits necroptosis but not apoptosis in Lm-infected mice.

### OTUB1 prevents necroptosis by stabilization of c-IAP1

**Fig. 5:**
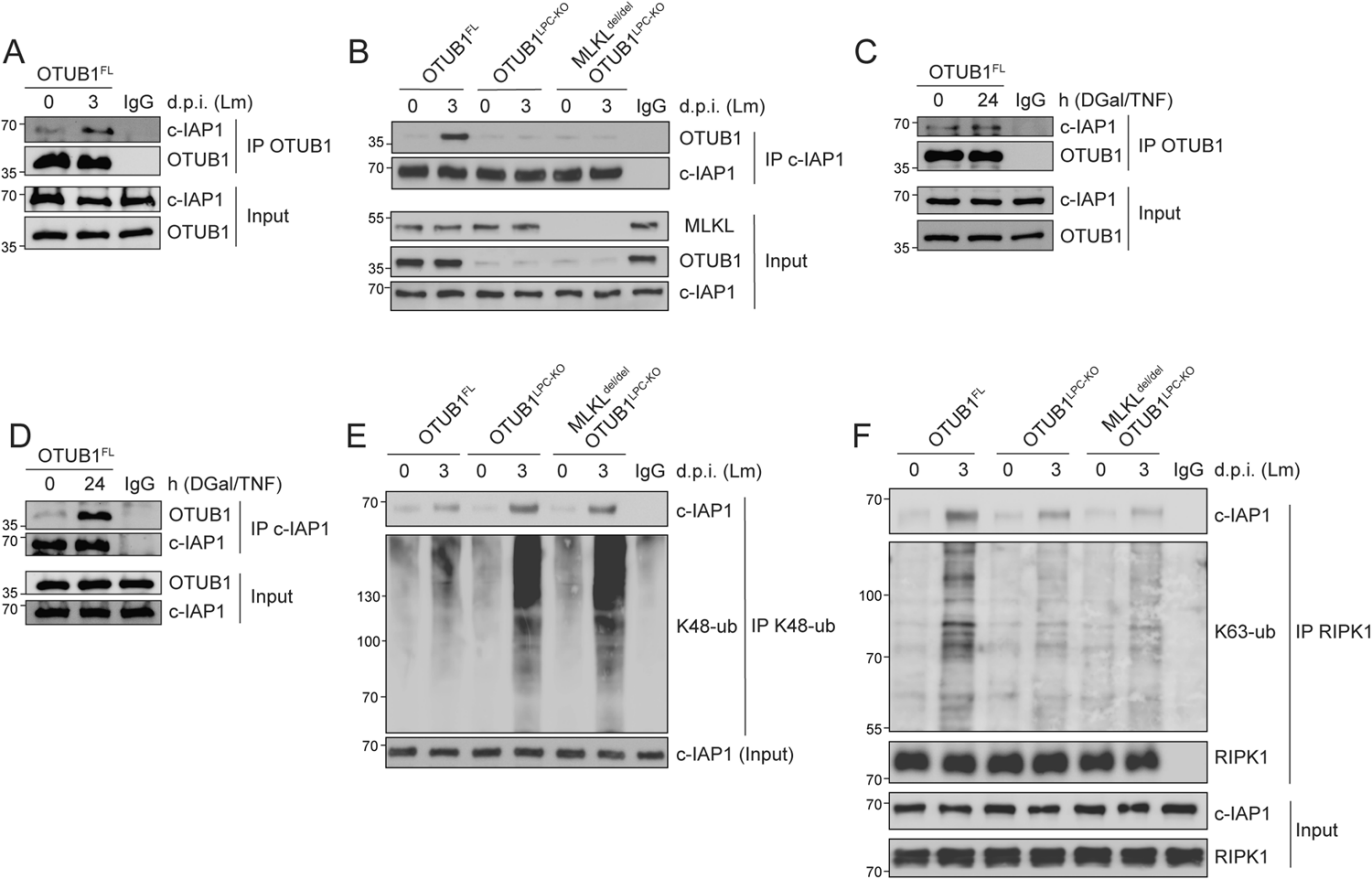
OTUB1 interacts with c-IAP1 and reduces K48-linked polyubiquitin of c-IAP1. OTUB1-c-IAP1 interactions were studied in liver tissue homogenates of unstimulated OTUB1^LPC-KO^, OTUB1^FL^, and MLKL^del/del^ OTUB1 ^LPC-KO^ mice as indicated upon **A**, **B** Lm infection (day 3 p.i.) and **C**, **D** DGal/TNF treatment (24 h). Protein complexes immunoprecipitated with either anti-OTUB1 (**A**, **C**) or anti-c-IAP1 (**B**, **D**) were immunoblotted with anti-OTUB1 and anti-c-IAP1, respectively. In these experiments, the c-IAP1 input was equalized between the experimental groups. **E** K48-linked polyubiquitination of c-IAP1 was analyzed in liver lysates of uninfected and Lm-infected mice (day 3 p.i.) by immunoprecipitating anti-ubiquitin (Lys48-specific). **F** Total proteins isolated from uninfected and Lm-infected OTUB1^LPC-KO^, OTUB1^FL^, and MLKL^del/del^ OTUB1 ^LPC-KO^ livers were immunoprecipitated with anti-RIPK1 and thereafter stained for RIPK1 and K63-linked polyubiquitination using anti-RIPK1 and anti-K63-ub. **A**–**F** Representative blots are displayed from one of three experiments each. c-IAP1 cellular inhibitor of apoptosis 1, Lm *Listeria monocytogenes*, p.i. post infection, d.p.i. days post infection, DGal D-Galactosamine, TNF tumor necrosis factor, RIPK receptor-interacting serine/threonine kinase, ub ubiquitin, IP immunoprecipitation.

Considering the increased in vivo degradation of c-IAP1 in the absence of OTUB1 (Fig. 3A, B), we studied whether OTUB1 protects hepatic c-IAP1 against proteasomal degradation in listeriosis and upon D-Gal/TNF treatment. Under both stimulatory conditions, c-IAP1 co-immunoprecipitated with OTUB1 and vice versa (Figs. 5A–D and S5A–D). In accordance to the deubiquitinase activity of OTUB1 on K48-linked polyubiquitin chains of c-IAP1 [21], we detected enhanced K48-linked polyubiquitination of c-IAP1 in the livers of OTUB1^LPC-KO^ mice upon *Listeria* infection, which was independent of MLKL (Figs. 5E and S5E). Upon TNF stimulation, c-IAP1 adds K63-linked polyubiquitin chains on RIPK1 which (i) serves as NF-κB activating signal and (ii) blocks its kinase activity required for RIPK1/RIPK3 necrosome formation [35]. Analysis of K63-linked polyubiquitination of RIPK1 in *Listeria*-infected livers revealed a strong K63-linked polyubiquitination of RIPK1 in OTUB1^FL^ mice but not in OTUB1^LPC-KO^ and MLKL^del/del^ OTUB1^LPC-KO^ mice (Figs. 5F and S5F). Collectively, these data illustrate that OTUB1 removes K48-linked polyubiquitin chains from c-IAP1 and reduces its proteasomal degradation. Hence, in OTUB1^FL^ mice, increased amounts of c-IAP1 resulted in enhanced K63-linked polyubiquitination of RIPK1, which inhibits RIPK1/RIPK3 necrosome formation.

### Hepatocyte-intrinsic function of OTUB1 protects from necroptosis and inhibits RIPK1/ERK-mediated TNF production

To extent our studies on the hepatocyte-intrinsic effect of OTUB1 on cell death pathways and TNF production, we analyzed Lm infection and TNF stimulation in human wildtype HepG2 cells, HepG2 cells with lentiviral deletion of OTUB1 and HepG2 cells with combined lentiviral OTUB1 and siRNA-mediated MLKL deletion.

**Fig. 6:**
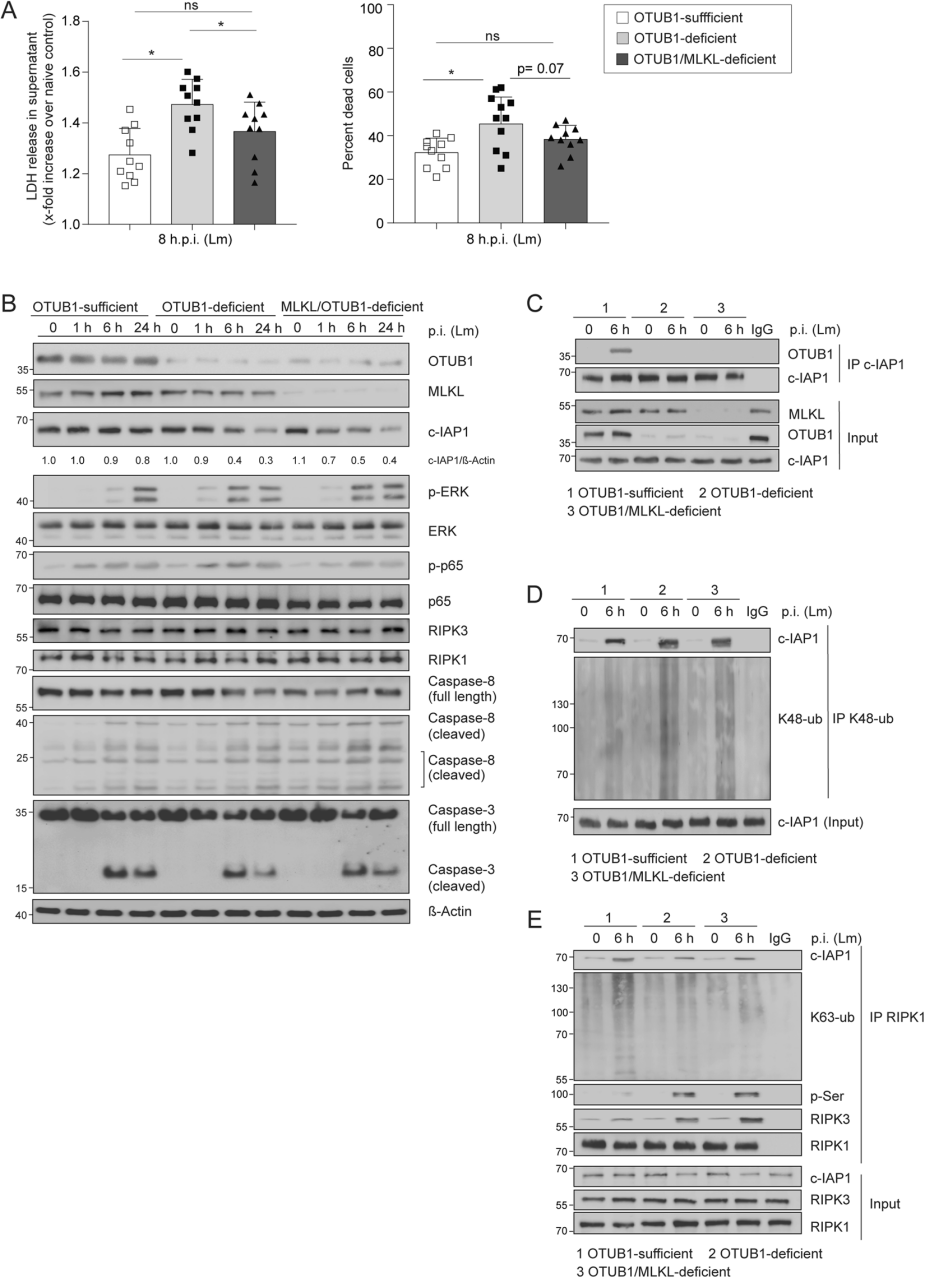
OTUB1 deletion in HEPG2 cells results in c-IAP1 destabilization and promotes necroptosis upon Lm-infection. **A**–**E** OTUB1 was stably knocked down in HepG2 cells using lentiviral transduction and MLKL was transiently deleted by siRNA treatment in the indicated OTUB1-deficient HepG2 cells. OTUB1-sufficient, OTUB1-deficient, and OTUB1/MLKL-deficient HepG2 cells were infected with Lm with a MOI of 10 for the indicated time points. **A** LDH release (left) was measured in the supernatant at 8 h.p.i. and calculated as x-fold increase over the respective uninfected controls (*n* = 10). The percentage of dead cells (right) were enumerated using trypan blue dye. **B** Cells were harvested at 0, 1, 6, and 24 h post Lm-infection, respectively, and immunoblotted with the indicated antibodies. Total proteins of uninfected and Lm-infected (6 h.p.i.) OTUB1-sufficient, OTUB1-deficient, and OTUB1/MLKL-deficient HepG2 cells were immunoprecipitated with anti-c-IAP1 (**C**), anti-ubiquitin (Lys48-specific) (**D**), and anti-RIPK1 (**E**), respectively, and analyzed by WB with the indicated antibodies. All blots are representative for one of three experiments. Lm *Listeria monocytogenes*, MOI multiplicity of infection, p.i. post infection, LDH lactate dehydrogenase, c-IAP1 cellular inhibitor of apoptosis 1, p phospho, ERK extracellular-signal-regulated kinase, IκB inhibitor of kappa B, RIPK receptor-interacting serine/threonine kinase, ub ubiquitin, p-Ser phospho-serine, WB western blot.

**Fig. 7:**
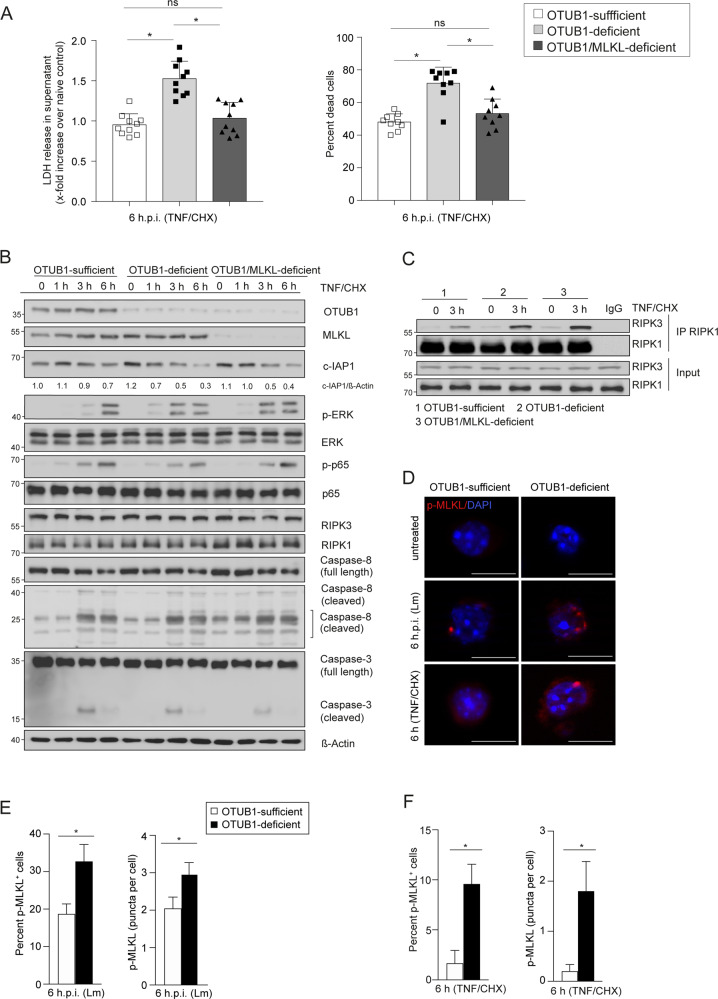
TNF stimulation triggers the degradation of c-IAP1 and necroptosis in OTUB1-deficient cells. **A**–**D** OTUB1-sufficient, OTUB1-deficient, and OTUB1/MLKL-deficient HepG2 cells were left untreated or stimulated with TNF (20 ng/ml)/CHX (10 µg/ml). **A** LDH release (left) was measured in the supernatant at 6 h and calculated as x-fold increase over the respective uninfected controls (*n* = 10). The percentage of dead cells (right) were enumerated using trypan blue staining. **B** OTUB1-sufficient, OTUB1-deficient, and OTUB1/MLKL-deficient HepG2 cells were left untreated or stimulated with TNF (20 ng/ml)/CHX (10 µg/ml) for 1, 3, and 6 h and, thereafter, analyzed by WB with the indicated antibodies. **C** RIPK1-associated complexes were analyzed in untreated and TNF (20 ng/ml)/CHX (10 µg/ml)-stimulated (3 h) HepG2 cells by IP. Representative blots from one of three experiments are shown. **D** OTUB1-sufficient and -deficient HepG2 cells were left untreated, infected with Lm for 6 h (MOI of 10) or stimulated with TNF (20 ng/ml)/CHX (10 µg/ml) for 6 h, respectively. Cells were stained with p-MLKL (red) and DAPI (blue) (×63, scale bars represent 20 µm). Representative immunofluorescence images are depicted. Numbers of p-MLKL^+^ cells and MLKL^+^ puncta per cell in Lm-infected (**E**) and TNF/CHX-stimulated (**F**) HepG2 cells were determined by counting 100 cells per group microscopically. Data show mean values + SEM. **D**, **E** Student’s *t* test, *p ≤ 0.05. CHX cycloheximide, p phospho, ERK extracellular-signal-regulated kinase, IκB Inhibitor of kappa B, RIPK receptor-interacting serine/threonine kinase, IP immunoprecipitation, MLKL mixed lineage kinase domain-like protein, Lm *Listeria monocytogenes*, h.p.i. hours post infection.

In agreement with the in vivo data, both expression of OTUB1 and deletion of MLKL in OTUB1-deficient HepG2 cells reduced LDH release upon infection with Lm (Fig. 6A) and stimulation with TNF (Fig. 7A). Likewise, the percentage of dead cells was also reduced in both Lm-infected and TNF-stimulated OTUB1-sufficient  and OTUB1/MLKL-deficient HepG2 cells, respectively (Figs. 6A and 7A). In addition, both Lm-infected and TNF-stimulated HepG2 cells showed that OTUB1 (i) diminished degradation of c-IAP1 (ii) reduced ERK phosphorylation and (iii) increased cleavage of caspase-3 and to a minor extent of caspase-8 but (iv) did not regulate activation of the NF-κB pathway (i.e. p65 phosphorylation) (Figs. 6B and 7B). The additional deletion of MLKL in OTUB1-deficient HepG2 cells did not change the increased c-IAP1 degradation and ERK activation, the reduced caspase-8 and -3 cleavage, and the normal NF-κB activation of OTUB1-deficient HepG2 cells (Figs. 6B and 7B). Furthermore, OTUB1 interacted with c-IAP1 (Fig. 6C) and inhibited K48-linked polyubiquitination of c-IAP1 (Fig. 6D) in Lm-infected cells. In Lm-infected HepG2 cells, RIPK1 interacted with c-IAP1, which was strongly reduced in OTUB1-deficient cells in an MLKL-independent manner (Fig. 6E). Since c-IAP1 mediates K63-linked polyubiquitination of RIPK1, which blocks the kinase activity of RIPK1 leading to necroptosis [32, 35, 36], we immunoprecipitated RIPK1 in Lm-infected OTUB1-sufficient, OTUB1-deficient and OTUB1/MLKL-deficient cells, respectively, and determined that OTUB1 (i) enabled RIPK1/c-IAP1 interaction, (ii) increased K63-linked polyubiquitination of RIPK1, (iii) inhibited serine phosphorylation of RIPK1, which is important for RIPK1-mediated cell death [35, 37] and reduced RIPK1 interaction with RIPK3 (Fig. 6E). Similarly, in TNF-stimulated HepG2 cells, OTUB1 reduced RIPK1/RIPK3 necrosome formation (Fig. 7C).

To substantiate that the enhanced RIPK1/RIPK3 interaction in Lm-infected and TNF-stimulated OTUB1-deficient HepG2 cells resulted in increased necroptosis, we stained for phospho-MLKL (p-MLKL). Both infection with Lm and stimulation with TNF significantly increased numbers of p-MLKL^+^ cells and the amount of p-MLKL per cell in OTUB1-deficient HepG2 cells as compared to OTUB1-sufficient HepG2 cells (Fig. 7D–F).

### OTUB1 inhibits RIPK1/ERK-dependent TNF production

**Fig. 8:**
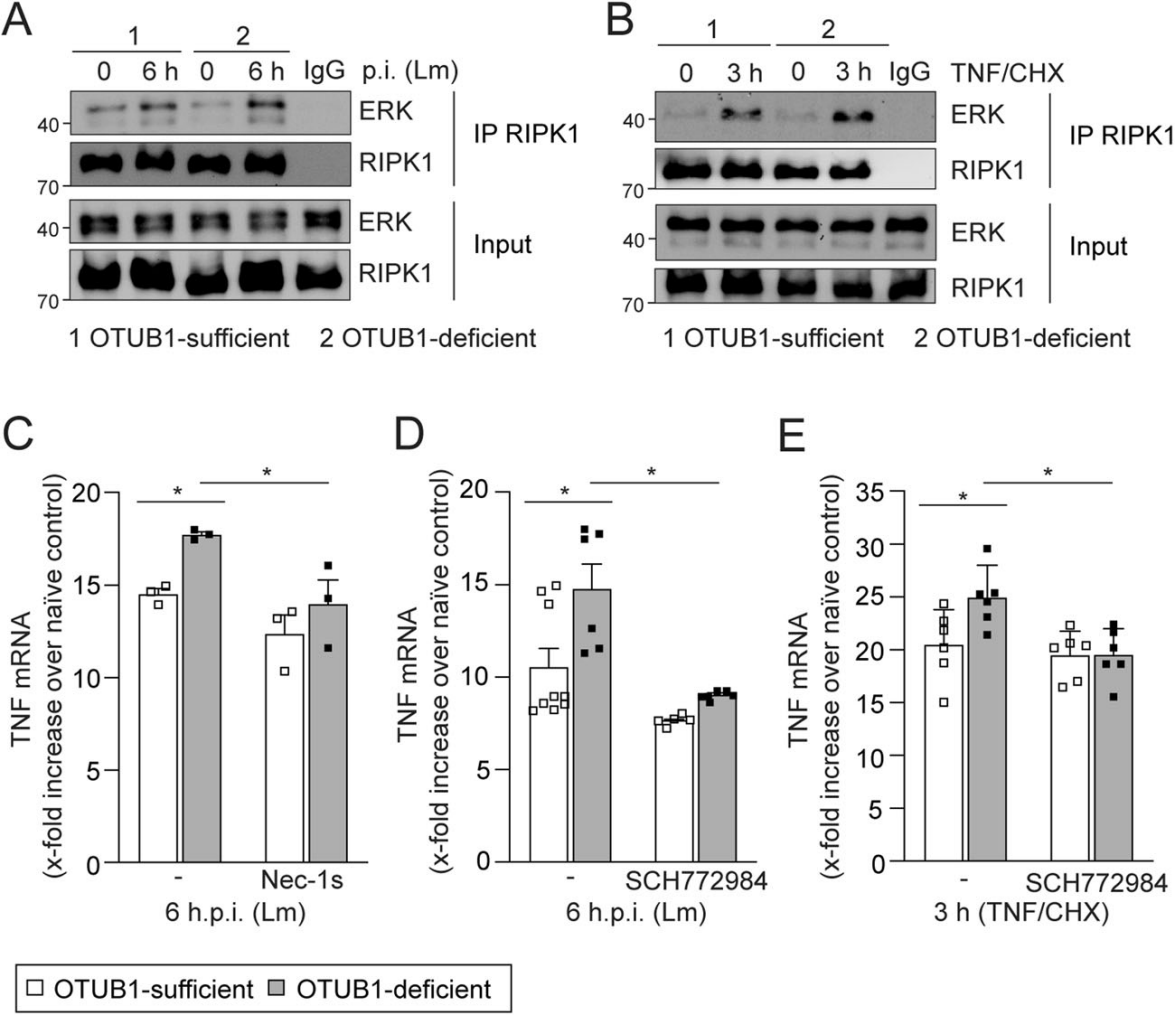
OTUB1 reduces RIPK1/ERK-dependent TNF expression. Cell lysates from untreated and Lm-infected (**A**) or uninfected and TNF (20 ng/ml)/CHX (10 µg/ml) stimulated (**B**) OTUB1-sufficient and -deficient HepG2 cells were immunoprecipitated using anti-RIPK1. RIPK1-complexes and inputs were probed with anti-RIPK1 and anti-ERK. **C** OTUB1-sufficient and -deficient HepG2 cells were left untreated or pretreated with Nec-1s (30 µM, 30 min) and subsequently infected with Lm (MOI of 10) for 6 h in the presence or absence of Nec-1s as indicated. At 6 h p.i., relative expression of TNF mRNA was quantified by qRT-PCR (*n* = 3). Quantitative analysis of TNF mRNA expression in Lm-infected (**D**) or TNF/CHX-stimulated (**E)** HepG2 cells with or without ERK inhibition. Treatment of HepG2 cells with the ERK inhibitor SCH772984 (0.5 µM) was started 30 min prior to the infection or stimulation (*n* = 6). Data of individual mice and the mean + SEM of the x-fold increase over the respective uninfected (**C**, **D**) and TNF-untreated (**E**) controls are shown. **C**–**E** two-way Anova, *p ≤ 0.05. Lm *Listeria monocytogenes*, CHX cycloheximide, RIPK receptor-interacting serine/threonine kinase, ERK extracellular-signal-regulated kinase, IP immunoprecipitation, MOI multiplicity of infection, p.i. post infection, qRT-PCR quantitative real-time PCR.

RIPK1 and RIPK3 can induce ERK-dependent TNF mRNA production in macrophages [38]. To determine whether OTUB1 regulates RIPK1/ERK-dependent TNF production, we stimulated HepG2 cells with Lm and TNF, respectively, and determined RIPK1/ERK interaction. Both Lm infection (Figs. 8A and S4B) and TNF stimulation (Fig. 8B) induced an interaction of RIPK1 with ERK independent of OTUB1 expression. Confirmatively, inhibition of RIPK1 by Nec-1s and ERK by SCH772984 significantly downregulated TNF mRNA expression in OTUB1-deficient cells and abolished differences between the two genotypes (Fig. 8C–E). Thus, OTUB1 inhibits hepatocyte necroptosis by two synergistic mechanisms, i.e. inhibition of the RIPK1/RIPK3/MLKL pathway and by the reduction of RIPK1/ERK-dependent TNF production, which further decreases TNF-induced necroptosis.

## Discussion

Inflammatory stimuli including TNF and pathogens can induce different types of cell death including apoptosis and necroptosis depending on the underlying cell type and the respective pathogen. With respect to hepatocytes, apoptosis but not necroptosis is induced in human and experimental diseases (reviewed in [3, 6, 39, 40]). Here, we identify OTUB1 as an inhibitor of hepatocyte necroptosis in listeriosis and upon TNF stimulation. The critical in vivo importance of OTUB1 as a hepatocyte-intrinsic inhibitor of necroptosis is illustrated by the lethal exacerbation of bacterial hepatitis and DGal/LPS-induced liver inflammation. Before, it has been demonstrated that inhibition or deletion of c-IAP1 and caspase-8, respectively, results in hepatocyte necroptosis and lethal hepatocyte damage further indicating that hepatocyte necroptosis is intrinsically inhibited to protect the liver [2].

So far it has been exemplified in listeriosis, that infected Kupffer cells undergo RIPK1/MLKL-dependent necroptosis, whereas hepatocytes may become apoptotic [11, 12 and this study]. This illustrates that cell-type-specific but not pathogen-specific factors account for the different forms of cell death in liver resident cells. Apoptosis of hepatocytes has been documented in various human diseases and experimental models including stimulation of mice with DGal/LPS and DGal/TNF, respectively [39, 40].

Under homeostatic conditions, interaction of OTUB1 and c-IAP1 was weak. However, infection with Lm and stimulation with TNF strongly increased association of OTUB1 with c-IAP1 and deubiquitination of K48-linked polyubiquitin chains from c-IAP1, thereby reducing its degradation. The reduction of c-IAP1 in OTUB1-deficient hepatocytes resulted in diminished apoptosis, induction of necroptosis and ERK-dependent TNF production upon infection with Lm and stimulation with TNF, respectively. This is in contrast to the rapid and complete degradation of c-IAP1 by c-IAP1 antagonists and activation of TWEAK-FN14 signaling, respectively, which both induce apoptosis but not necroptosis of tumor cell lines by NF-κB-dependent autocrine TNF-production [41–43]. These differences in c-IAP1 regulated cell death and pro-inflammatory signaling pathways may be explained by the distinct c-IAP1 levels and cell-type-specific effects, but potentially also by c-IAP1-independent effects of OTUB1 on other signaling molecules.

In TNFR1 signaling, stability of c-IAP1 in the TNFR1-signaling complex 1 is important for c-IAP1-dependent K11-, K48- and K63-linked polyubiquitination of RIPK1, which regulate the pro-survival function of RIPK1 in complex I and block the formation of the apoptosis and necroptosis inducing complexes II [44–46]. In particular, K63-linked polyubiquitination of RIPK1 is important to inhibit the kinase activity of RIPK1 and to prevent caspase-8 dependent apoptosis and RIPK3/MLKL-dependent necroptosis [32, 36]. Thus, in the absence of c-IAP1 and impaired RIPK1 ubiquitination, RIPK1 spontaneously triggers the formation of complex IIb, which can initiate both apoptosis and necroptosis [47]. In accordance, TNF stimulation of RIPK1 Lys376 (K376) mutant mouse embryonic fibroblasts, which exhibit impaired K63-linked polyubiquitination, undergo both RIPK1 kinase-dependent apoptosis and necroptosis, which was further enhanced in the presence of c-IAP inhibitors [32, 36]. In good agreement, the increased c-IAP1 degradation in Lm-infected OTUB1^LPC-KO^ mice and OTUB1-deficient HepG2 cells resulted in reduced K63-linked polyubiquitination of RIPK1 and cell death. In vivo, the critical function of the RIPK1 kinase activity for the exacerbation of listeriosis in OTUB1^LPC-KO^ mice is illustrated by the reduction of hepatocyte damage, cell death and prevention of lethal listeriosis upon inhibition of RIPK1 kinase activity by Nec-1s.

In addition to K63-linked polyubiquitination of RIPK1 by c-IAP1, linear M1 ubiquitination of RIPK1 by the linear-ubiquitin assembly complex (LUBAC) inhibits RIPK1-induced cell death upon TNF stimulation. LUBAC initiates recruitment of NEMO, IKKα/β, Tank-binding kinase-1 and IKKε to the TNFR1 signaling complex 1, and these kinases phosphorylate RIPK1 and, thereby, prevent cell death [48–52]. Of note, OTUB1 has only specificity for K48-linked but not for K63-linked and M1 ubiquitin chains [53] [27] and, thus, cannot directly reduce K63-linked and M1 ubiquitin-dependent phosphorylation of RIPK1. In contrast to the inhibition of RIPK1 kinase activity by LUBAC-dependent recruitment of RIPK1-phosporylating kinases, autophosphorylation of RIPK1 at serine 166 licenses RIPK1 kinase activity and cell death [37]. In this regard, we detected an increased total serine phosphorylation of RIPK1 in OTUB1-deficient Lm-infected HepG2 cells and it will be interesting to further explore how OTUB1 regulates the complex phosphorylation events of RIPK1 and its kinase activity.

In both Lm-infected and TNF-stimulated OTUB1-deficient mice and HepG2 cells, necroptosis induction was indicated by enhanced RIPK1/RIPK3-necrosome formation and phosphorylation of MLKL. Functionally important, in vivo inhibition of RIPK1 by Nec-1s completely prevented lethal listeriosis in OTUB1^LPC-KO^ mice and both Nec-1s treatment and combined OTUB1 and MLKL deletion significantly reduced liver damage in Lm-infected OTUB1^LPC-KO^ mice and cell death of Lm-infected and TNF-stimulated HepG2 cells, respectively. We also detected increased cleavage of caspase-8 and caspase-3 in vivo upon infection of OTUB1^FL^ mice with Lm and treatment with DGal/TNF, respectively. However, zVAD treatment of OTUB1^FL^ mice did not alter LDH and ALT levels in Lm infection indicating that hepatocyte apoptosis plays a minor role in the pathogenesis of listeriosis. Since caspase-8 inhibits RIPK1-mediated necroptosis [5], the partial cleavage of caspase-8 in combination with the simultaneously impaired K63-linked polyubiquitination of RIPK1, may be sufficient to induce necroptosis in OTUB1-deficient hepatocytes. This is in contrast to Goncharov et al., who demonstrated that destabilization of c-IAP1 in OTUB1-deficient endothelial and epithelial cells induced apoptosis upon in vitro activation of Fn14 by TWEAK. The reason for this discrepancy may relate not only to cell-type specificity but also to the different stimuli leading to divergent activation of pro-inflammatory signaling pathways. In TWEAK/FN14-activated OTUB1-deficient cells, c-IAP1 degradation impaired the activation of mitogen-activated protein kinase and canonical NF-κB pathways which sensitized these cells towards endogenously released TNF-dependent and caspases-mediated apoptosis [21]. In contrast, our study shows that OTUB1-deficiency in hepatocytes and the subsequent accelerated degradation of c-IAP1 (i) augmented Lm- and TNF-induced ERK activation without affecting canonical and noncanonical NF-κB activation and expression of NF-κB-dependent antiapoptotic genes (ii) induced RIPK1 and MLKL-dependent necroptosis. Functionally relevant, the increased RIPK1/ERK-dependent TNF mRNA production of OTUB1-deficient hepatocytes may support an autocrine feedback loop by binding of TNF to TNFR1 and fostering RIPK1-dependent necroptosis and TNF production. This is in accordance with data demonstrating that the RIPK1/RIPK3 necroptotic complex can associate with ERK to induce pro-inflammatory cytokines including TNF and that binding of TNF to the TNFR1 further amplifies necroptotic death. Collectively, OTUB1 is a hepatocyte-intrinsic factor inhibiting necroptosis upon bacterial infection and TNF-induced liver damage, respectively, thereby, protecting the host from lethal exacerbation of these inflammatory diseases.

## Supplementary information

Supplemental material
