# Correction: OTUB1 prevents lethal hepatocyte necroptosis through stabilization of c-IAP1 during murine liver inflammation

**DOI:** 10.1038/s41418-021-00819-7

**Published:** 2021-06-18

**Authors:** Josephin Koschel, Gopala Nishanth, Sissy Just, Kunjan Harit, Andrea Kröger, Martina Deckert, Michael Naumann, Dirk Schlüter

**Affiliations:** 1 Institute of Medical Microbiology and Hospital Epidemiology, Hannover Medical School, Hannover, Germany; 2 Institute of Medical Microbiology and Hospital Hygiene, Otto von Guericke University Magdeburg, Magdeburg, Germany; 3 Innate Immunity and Infection Group, Helmholtz Centre for Infection Research, Braunschweig, Germany; 4 Department of Neuropathology, Faculty of Medicine and University Hospital Cologne, University of Cologne, Cologne, Germany; 5 Institute of Experimental Internal Medicine, Otto von Guericke University Magdeburg, Magdeburg, Germany; 6 Cluster of Excellence RESIST (EXC 2155), Hannover Medical School, Hannover, Germany

**Keywords:** Cell death and immune response, Microbiology

Correction to: *Cell Death & Differentiation* 10.1038/s41418-021-00752-9

The original version of this article unfortunately contained a mistake in the affiliations. Since the publication of this article, the authors have noticed an error in the affiliation of the first author of the manuscript, Dr. Josephin Koschel. The correct affiliations are as follows:

Josephin Koschel 1, 5

1 Institute of Medical Microbiology and Hospital Epidemiology, Hannover Medical School, 30625 Hannover, Germany

5 Institute of Experimental Internal Medicine, Otto von Guericke University Magdeburg, 39120 Magdeburg, Germany

The authors apologize for the mistake. The original article has been corrected.

