# Supplementary material for: OTUB1 prevents lethal hepatocyte necroptosis through stabilization of c-IAP1 during murine liver inflammation
Source: Cell Death Differ. 2021 Mar 12;28(7):2257–75. doi: 10.1038/s41418-021-00752-9 (PMC8257688; doi:10.1038/s41418-021-00752-9)
Supplement: Supplementary file 1 — Supplemental material [file 41418_2021_752_MOESM1_ESM.docx]

Supporting information for:

**OTUB1 prevents lethal hepatocyte necroptosis through stabilization of c-IAP1 during murine liver inflammation**

**TABLE OF CONTENTS**

**SUPPLEMENTARY FIGURES……………………………………………………… 2**

**SUPPLEMENTARY TABLES……………………………………………………….. 10**

# SUPPLEMENTARY FIGURES


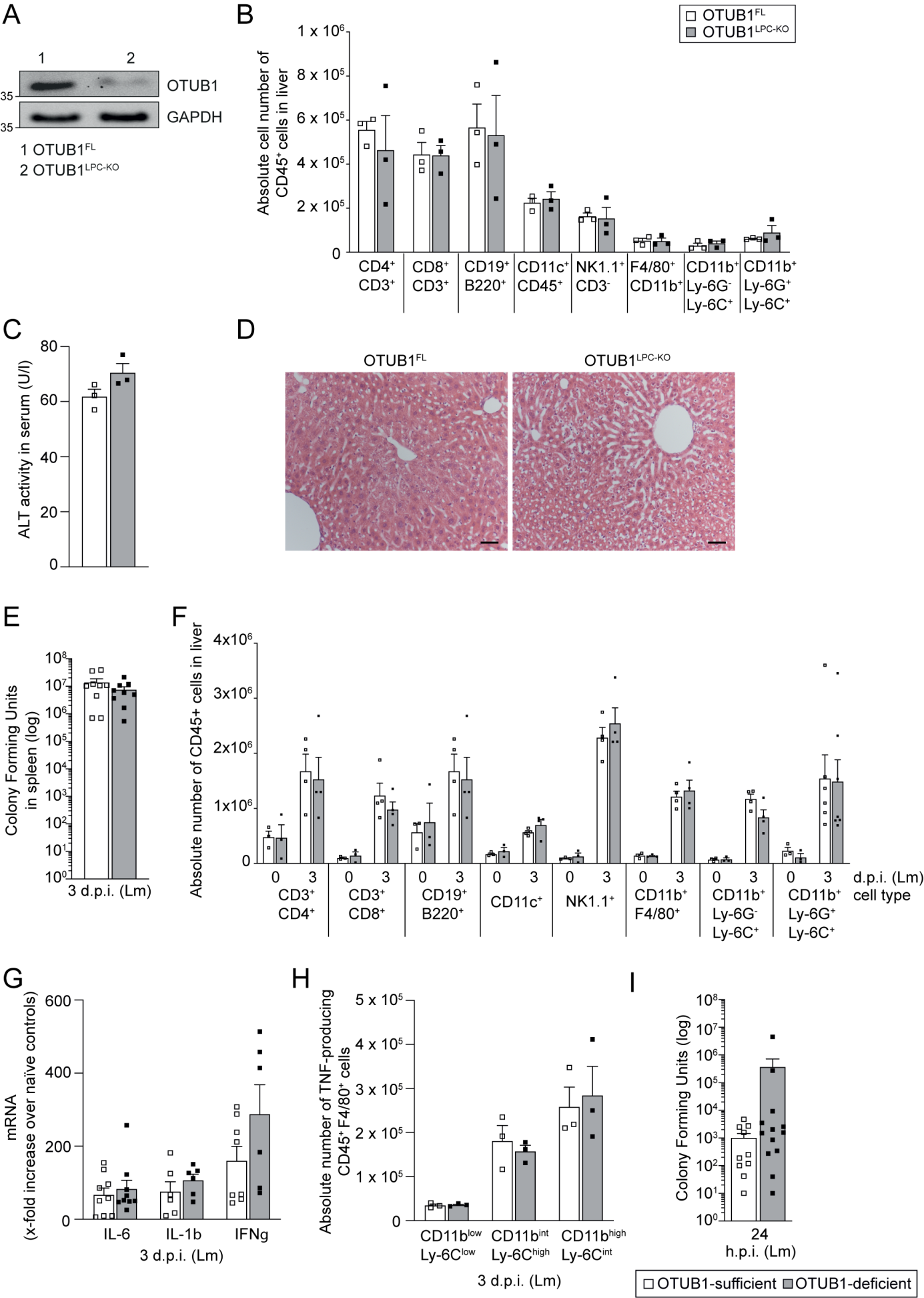


Supplementary Figure 1:

(A) Whole liver lysates from naïve OTUB1^LPC-KO^ and OTUB1^FL^ mice were analyzed for the expression of OTUB1 and GAPDH by WB. (B - D) Characterization of naïve one-year old OTUB1^LPC-KO^ and OTUB1^FL^ mice. (B) Absolute numbers of CD45^+^ subpopulations were analyzed in the liver by flow cytometry (n = 3). (C) ALT activity was measured in serum samples (n = 3 mice per group). (D) Livers sections from one-year old OTUB1^LPC-KO^ and OTUB1^FL^ mice were examined by histopathology. Representative images are depicted (H&E staining, bar = 50 µm, n = 3). (E) CFUs were determined in spleens at day 3 after i.v. infection with 5 x10^4^ CFU of Lm (n = 9 mice per group). (F) 3 days post Lm-infection, flow cytometric analysis for the hepatic CD45^+^ subpopulations were performed (uninfected: n = 3, infected n = 4 mice). (G) Relative gene expression of the indicated cytokines was quantified in the livers of uninfected and Lm-infected OTUB1^LPC-KO^ and OTUB1^FL^ mice by qRT-PCR at day 3 p.i. Data show the fold increase over the respective uninfected mouse strain (n = 6 - 9 mice per group). (H) CD45^+^ F4/80^+^ cells infiltrating to the liver at day 3 p.i. were characterized by flow cytometry for their TNF-production (n = 3 mice per group). (I) Primary hepatocytes isolated from OTUB1^LPC-KO^ and OTUB1^FL^ mice were infected with Lm with a MOI of 10. 24 h after infection, bacterial loads were determined (n = 10 – 13). Data of individual mice and the mean + SEM are displayed, (B, C, F - H) Student’s *t*-test, (E, I) Mann Whitney *U*-test, * p ≤ 0.05. Abbreviations: GAPDH, glyceraldehyde 3-phosphate dehydrogenase; CD, cluster of differentiation; WB, western blot; ALT, alanine aminotransferase; Lm, *Listeria monocytogenes*; CFU, colony forming units; H&E, haematoxylin & eosin; p.i. post infection; MOI, multiplicity of infection; h.p.i. hours post infection.


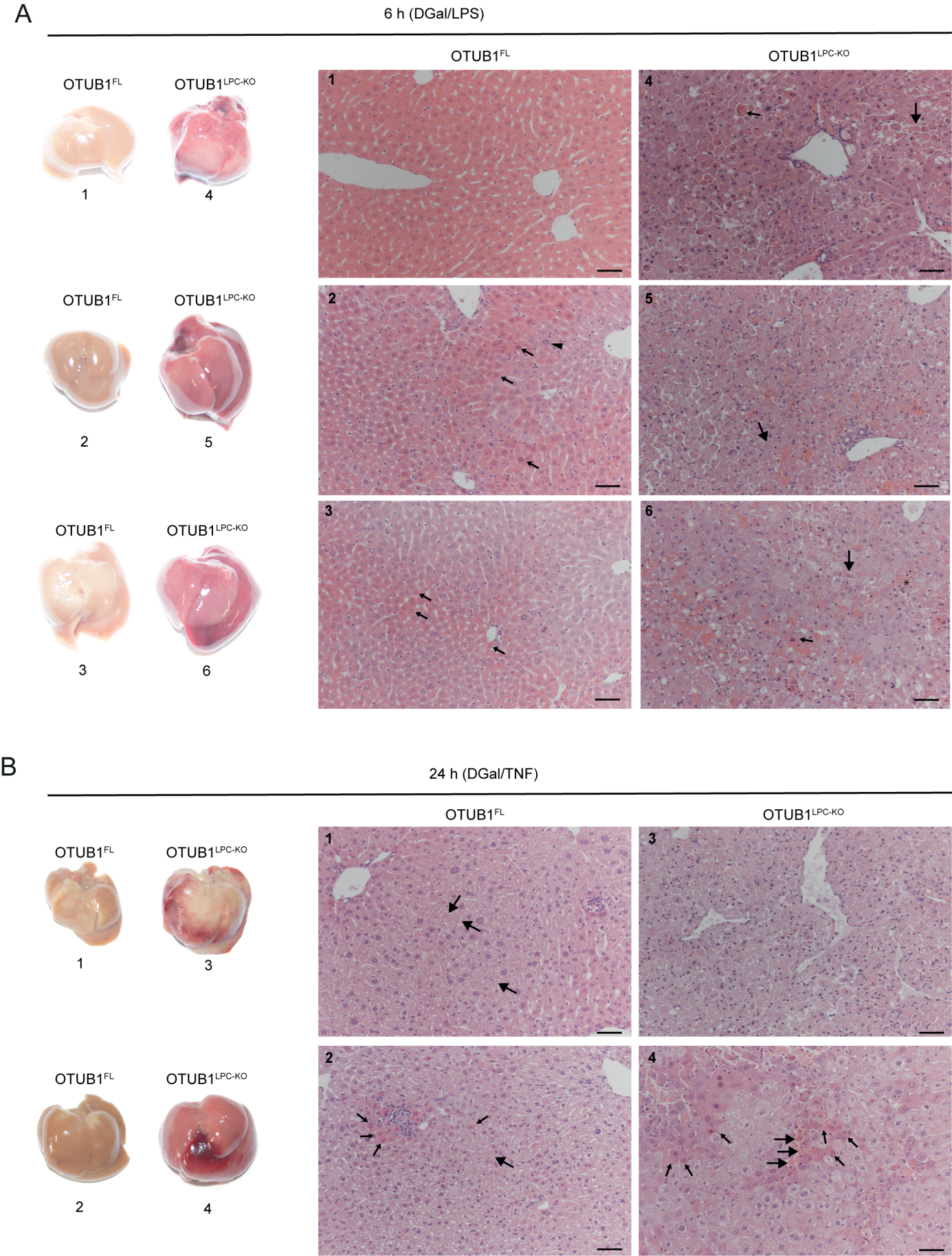


Supplementary Figure 2:

(A, B) The macroscopic pathology of DGal/LPS- (A) and DGal/TNF- (B) treated OTUB1^FL^ and OTUB1^LPC-KO^ mice is shown on the left side and the corresponding histology of the respectively numbered mice is shown on the right side. (A) Six hours after DGal/LPS treatment, livers of OTUB^FL^ and OTUB1^LPC-KO^ mice livers were isolated. Macroscopically, livers of OTUB^FL^ are normal. Histology shows also a normal liver architecture (no. 1) or only minor damage (no. 2, 3) with a mild cytoplasmic eosinophilia (no. 2, 3; arrows) and occasionally swollen nuclei of hepatocytes (no. 2, arrowhead). In contrast, OTUB^LPC-KO^ mice uniformly exhibit severe macroscopic hepatic damage with hemorrhages (no. 4 - 6). Microscopically, the tissue has disintegrated with lack of hepatic architecture (no. 4 - 6). Many hepatocytes have already lost their nuclei (no. 4 - 6; large arrows). Additionally, swollen but also shrunken hepatocytes with a prominently eosinophilic cytoplasm and nuclear fragments are present (no. 4, 6; small arrows). Multiple foci of acute hepatic hemorrhage are discernable (no. 6, left to *). (B) Six hours after DGal/TNF challenge, the liver of OTUB1^FL^ mice shows macroscopically a normal pale color (1, 2). Histologically, OTUB^FL^ mice show a disturbed hepatic architecture and some hepatocytes have lost their nuclei and show a coarse, pale, weakly eosinophilic cytoplasm (no. 1, 2; large arrows). Additionally, vital hepatocytes are swollen and show increased cytoplasmic eosinophilia (no. 2, small arrows). In contrast, OTUB1^LPC-KO^ mice show macroscopically a severe liver pathology with hemorrhages (no. 3, 4). Many hepatocytes of OTUB1^LPC-KO^ mice exhibit cytoplasmic eosinophilia and swollen nuclei (no. 4, small arrows). In part, hepatocytes have lost their nuclei (no. 4, large arrows). In one OTUB^LPC-KO^ mouse (no. 3), the hepatic architecture is severely disintegrated with wide areas without discernible hepatocytes. (A, B) H&E staining, scale bars indicate 50 µm. Abbreviations: DGal, D-Galactosamine; LPS, Lipopolysaccharide; TNF, tumor necrosis factor; H&E, haematoxylin and eosin.


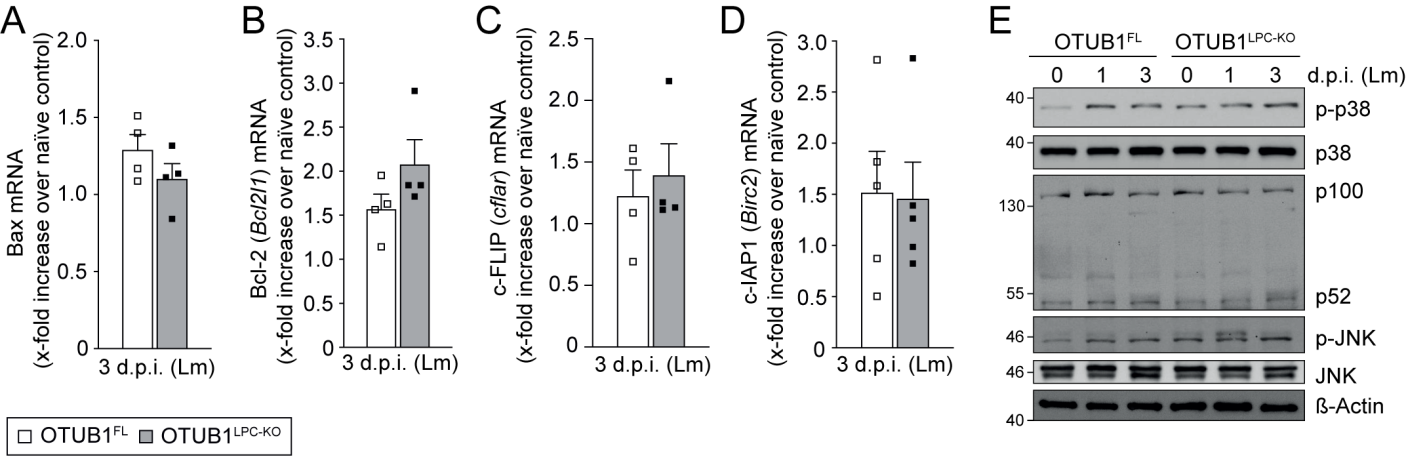


Supplementary Figure 3:

(A - D) Day 3 post i.v. infection with 5 x 10^4^ CFU of Lm, the relative expression of the indicated genes was quantified in liver tissue from OTUB1^LPC-KO^ and OTUB1^FL^ mice at day 3 qRT-PCR (n = 4 mice per goup). Data of individual mice and mean values + SEM are depicted, Student’s *t*-test, * p ≤ 0.05. (E) WB analysis of p-p38, p38, p100, p52, p-JNK, JNK and β-Actin in protein lysates isolated from liver tissue of uninfected and Lm-infected OTUB1^LPC-KO^ and OTUB1^FL^ mice at day 1 and day 3 p.i., respectively. Blots are representative for one of three mice per group. Abbreviations: Lm, *Listeria monocytogenes*; CFU, colony forming units; Bcl-2, B cell lymphoma-2; Bax, Bcl-2-associated X protein; c-FLIP, cellular FLICE-like inhibitory protein; c-IAP1, cellular inhibitor of apoptosis; qRT-PCR, quantitative real-time polymerase chain reaction; WB, western blot; JNK, c-Jun N-terminal kinase; p.i., post infection; i.v., intravenous.


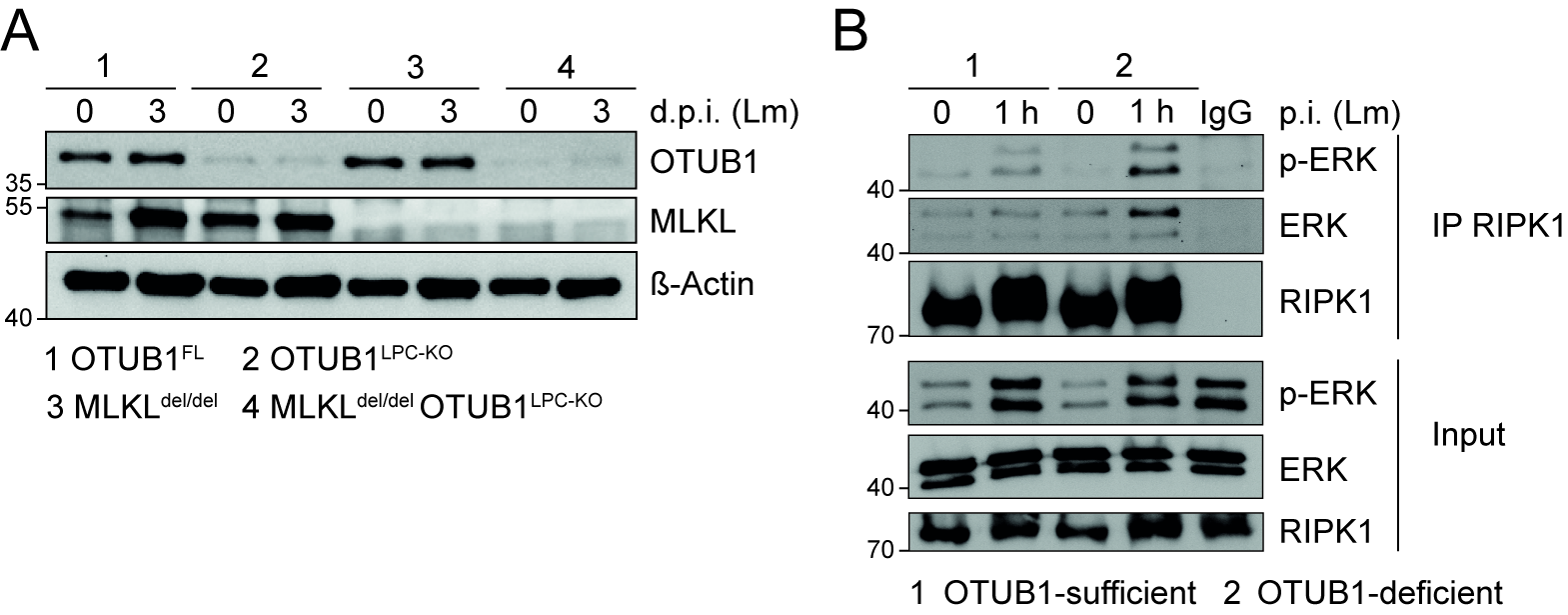


Supplementary Figure 4:

(A) WB analysis of OTUB1, MLKL and β-Actin expression in whole liver lysates obtained from OTUB1^FL^, OTUB^LPC-KO^, MLKL^del/del^, MLKL^del/del^ OTUB1^LPC-KO^ mice. (B) OTUB1-sufficient and -deficient HepG2 cells were left untreated or infected with Lm (MOI of 10) for 60 min. Protein lysates were harvested, immunoprecipitated using anti-RIPK1 and immunoblotted with the respective antibodies for RIPK1, ERK and p-ERK. All blots are representatives. Abbreviations: MLKL, mixed lineage kinase domain-like protein; Lm, *Listeria monocytogenes*; CFU, colony forming units; p.i., post infection; MOI, multiplicity of infection; RIPK, receptor-interacting serine/threonine kinase; p, phospho; ERK, extracellular-signal regulated kinase;IP, immunoprecipitation; d.p.i., days post infection; h, hour.

**Supplementary Figure 5**


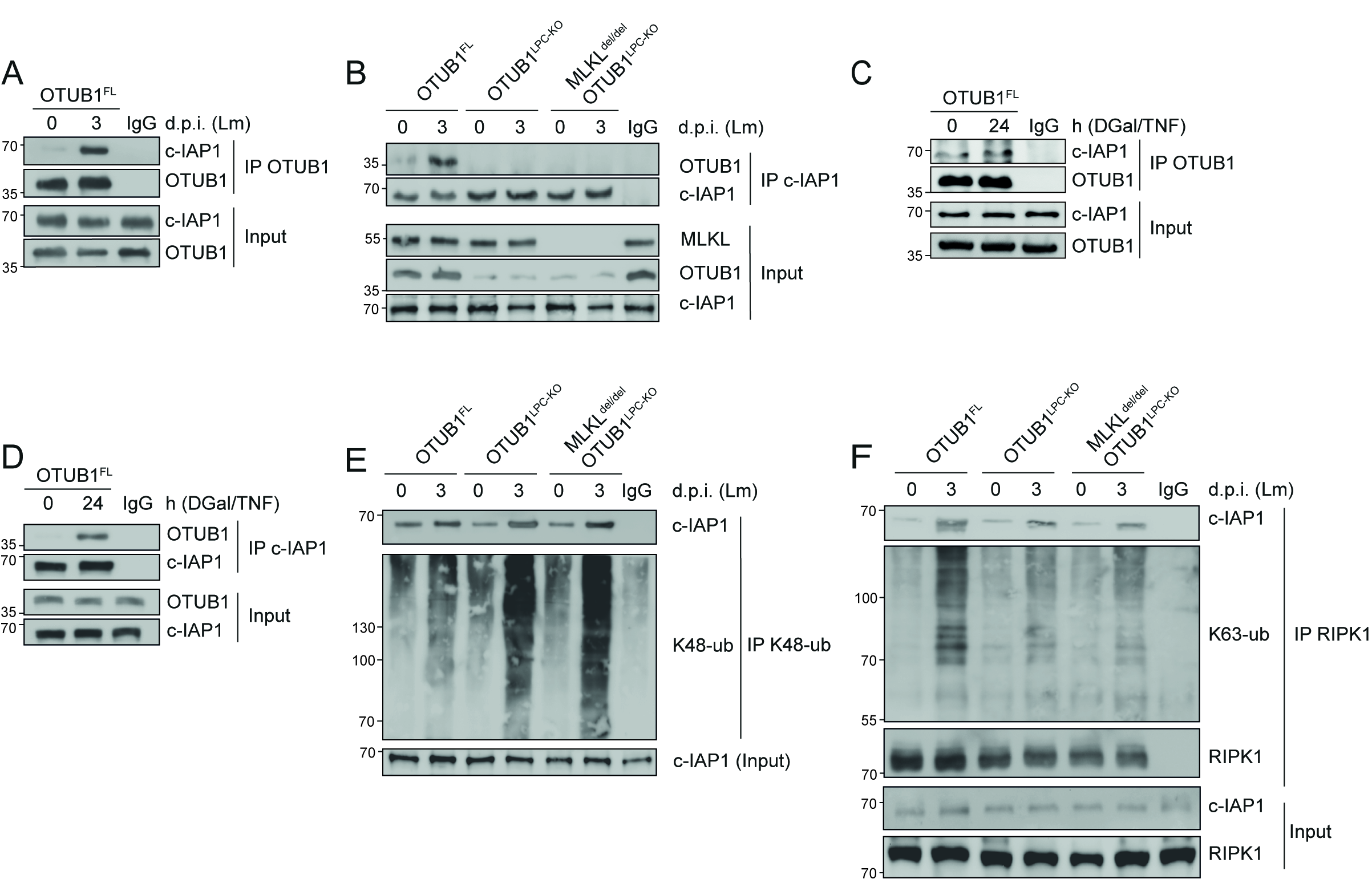


**Supplementary Figure 5:**

(A - D) OTUB1-c-IAP1 interactions were studied in liver tissue homogenates of unstimulated OTUB1^LPC-KO^, OTUB1^FL^ and MLKL^del/del^ OTUB1^LPC-KO^ mice as indicated upon (A, B) Lm infection (day 3 p.i.) and (C, D) DGal/TNF treatment (24 h). Protein complexes immunoprecipitated with either anti-OTUB1 (A, C) or anti-c-IAP1 (B, D) were immunoblotted with anti-OTUB1 and anti-c-IAP1, respectively. In these experiments, the c-IAP1 input was equalized between the experimental groups. (E) K48-linked polyubiquitination of c-IAP1 was analyzed in liver lysates of uninfected and Lm-infected mice (day 3 p.i.) by immunoprecipitating anti-ubiquitin (Lys48-specific). (F) Total proteins isolated from uninfected and Lm-infected OTUB^LPC-KO^, OTUB1^FL^ and MLKL^del/del^ OTUB1^LPC-KO^ livers were immunoprecipitated with anti-RIPK1 and thereafter stained for RIPK1 and K63-linked polyubiquitination using anti-RIPK1 and anti-K63-ub. (A - F) Representative blots are displayed from one of three experiments each. Abbreviations: c-IAP1, cellular inhibitor of apoptosis 1; Lm, Listeria monocytogenes; p.i., post infection; d.p.i., days post infection; DGal, D-Galactosamine; TNF, tumor necrosis factor; RIPK, receptor-interacting serine/threonine kinase; ub, ubiquitin; IP, immunoprecipitation.

# SUPPLEMENTARY TABLES

**Supplementary Table 1: TaqMan probes for quantitative real-time PCR**

| Gene | Assay ID |
| --- | --- |
| *Bax* | Mm00432051 |
| *Bcl2l1* | Mm00437783 |
| *Cflar* | Mm01255576 |
| *Birc2* | Mm00431811 |
| *Hprt* | Mm01545399 |
| *Ifng* | Mm00801778 |
| *Il1b* | Mm00434228 |
| *Il6* | Mm00446190 |
| *Tnf* | Mm00443258 |

Supplementary Table 2: Fluorochrome-conjugated antibodies for flow cytometric analyses.

| **Antibody** | **Cat#** | **Manufacturer** |
| --- | --- | --- |
| APC anti-mouse CD11c | 117309 | Biolegend (San Diego, USA) |
| APC anti-mouse Ly-6C | 17-5932-82 | eBioscience by affymetrix  (San Diego, USA) |
| APC anti-mouse TNF | 506307 | Biolegend (San Diego, USA) |
| APC anti-mouse IgG1, κ | 400411 | Biolegend (San Diego, USA) |
| APC-Cy7 anti-mouse CD11b | 101225 | Biolegend (San Diego, USA) |
| BV421 anti-mouse CD45R/B220 | 562922 | BD Biosciences (San Jose, USA) |
| BV421 anti-mouse F4/80 | 123137 | Biolegend (San Diego, USA) |
| BV510 anti-mouse CD3 | 563024 | BD Biosciences (San Jose, USA) |
| BV510 anti-mouse CD8 | 100752 | Biolegend (San Diego, USA) |
| BV510 anti-mouse CD45 | 103137 | Biolegend (San Diego, USA) |
| FITC anti-mouse Ly-6G | 127605 | Biolegend (San Diego, USA) |
| FITC anti-mouse CD3 | 11-0031-85 | eBioscience by affymetrix  (San Diego, USA) |
| FITC anti-mouse CD4 | 553046 | BD Biosciences (San Jose, USA) |
| PE anti-mouse CD19 | 12-0191-85 | eBioscience by affymetrix  (San Diego, USA) |
| PE anti-mouse NK 1.1 | 553165 | BD Biosciences (San Jose, USA) |
| PeCy7 anti-mouse CD3 | 100319 | Biolegend (San Diego, USA) |
| PeCy7 anti-mouse CD11b | 101215 | Biolegend (San Diego, USA) |
| PerCP anti-mouse CD45 | 103129 | Biolegend (San Diego, USA) |
| PerCP/Cy5.5 anti-mouse Ly-6C | 128011 | Biolegend (San Diego, USA) |

Supplementary Table 3: Primary antibodies for western blotting and immunoprecipitation

| **Antibody** | **Cat#** | **Manufacturer** |
| --- | --- | --- |
| Anti-ß-Actin | 8457 | Cell Signaling Technologies (Danvers, USA) |
| Anti-Bax | NBP1-28566 | Novus Biologicals (Centennial, USA) |
| Anti-Bcl-2 | 2876 | Cell Signaling Technologies (Danvers, USA) |
| Anti-BID | 2003 | Cell Signaling Technologies (Danvers, USA) |
| Anti-BID Cleavage Site  Anti-Caspase-3  Anti-Caspase-6  Anti-Caspase-7 | ab10640  9762  12827  9662 | Abcam (Cambridge, UK)  Cell Signaling Technologies (Danvers, USA)  Cell Signaling Technologies (Danvers, USA)  Cell Signaling Technologies (Danvers, USA) |
| Anti-Caspase-8 | 9429  4790  9746 | Cell Signaling Technologies (Danvers, USA) |
| Anti-c-IAP1 | ab154525 | Abcam (Cambridge, UK) |
| Anti-ERK1/2 | 9102 | Cell Signaling Technologies (Danvers, USA) |
| Anti-IκBα | 4812 | Cell Signaling Technologies (Danvers, USA) |
| Anti-p-IκBα | 2859 | Cell Signaling Technologies (Danvers, USA) |
| Anti-K63 TUBE  Anti-MLKL | UM304  604 | LifeSensors (Malvem, USA)  Merck Millipore (Darmstadt, Germany) |
| Anti-OTUB1 | NBP1-49934 | Novus Biologicals (Centennial, USA) |
| Anti-p-ERK1/2 | 9101 | Cell Signaling Technologies (Danvers, USA) |
| Anti-p-p38 | 9215 | Cell Signaling Technologies (Danvers, USA) |
| Anti-p-p65 | 3033 | Cell Signaling Technologies (Danvers, USA) |
| Anti-p-JNK | 9251 | Cell Signaling Technologies (Danvers, USA) |
| Anti-p100/52 | 4882 | Cell Signaling Technologies (Danvers, USA) |
| Anti-p38 | 9212 | Cell Signaling Technologies (Danvers, USA) |
| Anti-p65 | 8242 | Cell Signaling Technologies (Danvers, USA) |
| Anti-RIPK1 | 3493 | Cell Signaling Technologies (Danvers, USA) |
| Anti-RIPK3 | ab152130 | Abcam (Cambridge, UK) |
| Anti-JNK  Anti-TRAF2 | 9252  4724 | Cell Signaling Technologies (Danvers, USA)  Cell Signaling Technologies (Danvers, USA) |
| Anti-ubiquitin  (Lys48-Specific) | 05-1307 | Merck Millipore (Darmstadt, Germany) |
